# Hemodynamic Response Function in Brain White Matter in a Resting State

**DOI:** 10.1093/texcom/tgaa056

**Published:** 2020-08-28

**Authors:** Ting Wang, D Mitchell Wilkes, Muwei Li, Xi Wu, John C Gore, Zhaohua Ding

**Affiliations:** Department of Computer Science, Chengdu University of Information Technology, Chengdu, Sichuan 610225, China; Institute of Imaging Science, Vanderbilt University, Nashville, TN 37232, USA; Department of Electrical Engineering & Computer Science, Vanderbilt University, Nashville, TN 37232, USA; Institute of Imaging Science, Vanderbilt University, Nashville, TN 37232, USA; Department of Radiology and Radiological Sciences, Vanderbilt University Medical Center, Nashville, TN 37232, USA; Department of Computer Science, Chengdu University of Information Technology, Chengdu, Sichuan 610225, China; Institute of Imaging Science, Vanderbilt University, Nashville, TN 37232, USA; Department of Radiology and Radiological Sciences, Vanderbilt University Medical Center, Nashville, TN 37232, USA; Department of Biomedical Engineering, Vanderbilt University, Nashville, TN 37212, USA; Institute of Imaging Science, Vanderbilt University, Nashville, TN 37232, USA; Department of Electrical Engineering & Computer Science, Vanderbilt University, Nashville, TN 37232, USA; Department of Biomedical Engineering, Vanderbilt University, Nashville, TN 37212, USA

**Keywords:** BOLD, fMRI, hemodynamic response function, resting state, white matter

## Abstract

The hemodynamic response function (HRF) characterizes temporal variations of blood oxygenation level-dependent (BOLD) signals. Although a variety of HRF models have been proposed for gray matter responses to functional demands, few studies have investigated HRF profiles in white matter particularly under resting conditions. In the present work we quantified the nature of the HRFs that are embedded in resting state BOLD signals in white matter, and which modulate the temporal fluctuations of baseline signals. We demonstrate that resting state HRFs in white matter could be derived by referencing to intrinsic avalanches in gray matter activities, and the derived white matter HRFs had reduced peak amplitudes and delayed peak times as compared with those in gray matter. Distributions of the time delays and correlation profiles in white matter depend on gray matter activities as well as white matter tract distributions, indicating that resting state BOLD signals in white matter encode neural activities associated with those of gray matter. This is the first investigation of derivations and characterizations of resting state HRFs in white matter and their relations to gray matter activities. Findings from this work have important implications for analysis of BOLD signals in the brain.

## Introduction

Blood oxygenation level-dependent (BOLD) signals have been widely used to measure neural activity and functional connectivity in the human brain with functional magnetic resonance imaging (fMRI) (Ogawa et al. 1990). A major paradigm of fMRI is based on resting state acquisitions (Biswal et al. 1995), using which several “resting state networks” (RSNs) in cortical gray matter (GM) have been reliably detected (Yeo et al. 2011). One such component is the default mode network (DMN) whose central node resides in the posterior cingulate cortex (PCC), and which is considered responsible in part for intrinsic awareness during rest but is deactivated under attention demanding tasks (Raichle 2015).

Although spontaneous neural activities in GM have been the primary focus of resting state fMRI acquisitions, corresponding resting state BOLD fluctuations in the signals from white matter (WM) have usually been ignored. However, considerable evidence of reliable detections of BOLD signals in WM has accumulated, particularly in response to explicit tasks or stimulations (see ref Grajauskas et al., 2019 for review). In a recent tactile stimulation study, Wu et al. (2017) found that fiber bundles in the somatosensory circuit exhibited significantly greater temporal correlations with the primary sensory cortex and greater signal power during tactile stimulations than in a resting state. In a visual stimulation study, Huang et al. (2018) demonstrated that specific WM regions are robustly activated, which includes the optic radiations (ORs) and other structures related to visual activity. In the meantime, resting state BOLD signals in WM have also been increasingly recognized. For example, temporal correlations of low frequency resting state signals that persisted over long distances within distinct WM structures were reported and shown to be anisotropic by Ding et al. (2013). By clustering BOLD signals acquired in a resting state, Peer et al. (2017) found that symmetrical WM functional networks could be identified, and were correlated specifically with functional networks in GM. Marussich et al. (2017) used independent components analysis to derive spatially independent patterns within WM that appeared to reorganize during natural vision. More recently, differential correlation patterns between WM tracts and GM parcels were quantified in a resting state, and these were altered in response to visual stimulations (Ding et al. 2018). Taken together, these studies suggest that BOLD signals in WM in a resting state also reflect neural activities associated with GM.

Given the growing evidence for the existence of BOLD signals in WM, there remain unanswered questions regarding the nature of the hemodynamic response function (HRF) in WM. By means of event-related paradigms, we have observed that the HRF in WM may be characterized by a reduced peak amplitude and delayed peak time relative to GM (Li et al. 2019). Similar observations were also made earlier by a few other groups using functional tasks or CO_2_ challenges. For instance, Yarkoni et al. (2009) reported that certain WM regions exhibited delayed and subdued hemodynamic responses compared to GM in a reaction-time based fMRI analysis, and Fraser et al. (2012) found a reduced peak amplitude in WM responses to visual stimulations. Meanwhile, Courtemanche et al. (2018) and Tae et al. (2014) both found delayed functional activities in the corpus callosum. The time delay appeared more pronounced during a hypercapnic challenge in which, compared with GM, a substantially slower response was observed in WM (Rostrup et al. 2000), a phenomenon that was also seen in experiments of cerebrovascular reactivity to CO_2_ inhalation (Thomas et al. 2014).

Although task-based and event-related studies have yielded valuable insights into the HRF profiles in specific WM tracts, it is unclear in general how WM BOLD signal variations in a resting condition are modulated in time, which tends to be of more fundamental interest and is independent of the nature of the task or stimulus. However, the lack of explicit event onset times during a resting state presents a significant challenge to the derivation of HRFs. Previously, there have been observations of avalanches in brain activity (Beggs and Plenz 2003; Liu and Duyn 2013), which are presumably intrinsic spontaneous events that evoke transient hemodynamic responses in the brain. By using activity avalanches in GM as references, and assuming associations of BOLD signals in WM with neural activities in GM, HRFs in WM under resting conditions may be derived.

This study was motivated to extract HRFs in WM on the basis of activity avalanches and characterize their profiles in resting state fMRI. We demonstrate that HRFs in WM can be derived using neural activity avalanches in GM as synchronizing events, and the derived HRFs have reduced peak amplitudes and delayed peak times compared to the usual canonical waveform assumed for GM.

GM avalanches with smaller amplitudes were associated with reduced amplitudes in the derived WM HRFs, and the derived amplitudes tended to approach zero when the reference time points were arbitrarily chosen. By mapping the HRF latencies and signal correlations between WM and GM references, we find that the distribution patterns of signal latencies as well as temporal correlations show regional specificity to GM. Moreover, we used diffusion tractography to reconstruct WM tracts connecting to GM reference regions, and demonstrated that there was strong correspondence between tract structure and signal lags.

## Materials and Methods

### Subjects

The present study was approved by the Vanderbilt University Institutional Review Board. Thirty-two healthy and right-handed individuals (16 M/16F; mean age, 33.03 ± 11.1; range, 21–55) with no history of neurological or psychiatric disorders were recruited. Written informed consents were obtained from all subjects.

### Image Acquisitions

Imaging was performed on a 3T MRI scanner (Philips Healthcare, Inc., Best, Netherlands) with a 32-channel head array coil at Vanderbilt University Institute of Imaging Science. Functional MRI of each subject included 1 run of 200 volumes acquired using a T_2_*-weighted single-shot gradient echo (GE), echo-planar imaging (EPI) sequence with a repetition time (TR) of 2 s (total acquisition time = 400 s). Each functional volume consisted of 34 axial slices with the following parameters: spatial resolution = 3 × 3 × 3.5 mm^3^; SENSE factor = 2; matrix size = 80 × 80; field of view (FOV) = 240 × 240 mm^2^; echo time = 35 ms; slice gap = 0.5 mm. To provide anatomical references, high-resolution T_1_-weighted images were also acquired using a multishot, 3D magnetization-prepared rapid GE sequence at voxel size of 1 × 1 × 1 mm^3^. During the image acquisitions, subjects lay in a supine position with eyes closed in a resting state. Restricting pads within the head coil were used to minimize potential subjects’ head motion during the process of image acquisitions.

For reconstructions of WM tracts, diffusion weighted images (DWIs) were acquired using an EPI sequence and 32 diffusion-encoding directions (acquisition parameters: b = 1000 s/mm^2^, TR = 4500 ms, TE = 84 ms, matrix size = 112 × 112, FOV = 240 × 240 mm^2^, voxel size = 2 × 2 mm^2^, slice thickness = 2.0 mm, slice gap = 0).

### FMRI Preprocessing

All images were preprocessed using the statistical parametric mapping (SPM12) software package (www.fil.ion.ucl.ac.uk/spm/software/spm12). First, the functional images for each participant were slice-timing corrected and realigned to the mean volume using a 6-parameter rigid body transformation, which were subsequently corrected for head motion. Based on the motion parameters from the SPM12, subjects with maximum head translation >2 mm or rotation >2° within the 200 volumes were excluded. Second, for each subject, the T_1_ structural image was segmented to obtain the GM, WM, and cerebrospinal fluid (CSF) masks, which were coregistered to the mean fMRI volume from the same subject and then spatially normalized to the Montreal Neurological Institute (MNI) space. Finally, linear trends were removed from the normalized fMRI time series to correct for signal drifting, followed by temporal filtering using a low-pass filter at *f* < 0.1 Hz. To further reduce the effects of confounding factors that might contribute to artificial correlations in fMRI time series, we also removed several sources of spurious variance by linear regressions, including 6 head motion parameters and average signals from CSF according to a previous fMRI study (Behzadi et al. 2007).

### Extraction of GM and WM BOLD Signals

The analyses in this study were restricted to GM and WM regions. To begin with, a mean WM mask was first defined by averaging individual WM segments from the study subjects and then thresholded at a value of >0.8, so as to minimize potential partial volume effects from adjacent GM voxels. A similar process was carried out to define a mean GM mask based on individual GM segments with a threshold value of 0.6. The thresholded mean WM and GM masks were used as common masks for each of the subjects studied. Next, GM was parcellated into 90 structures (45 in each hemisphere, cerebellum excluded) according to the Automated Anatomic Labeling (AAL) atlas (Tzourio-Mazoyer et al. 2002). These GM regions of interest (ROIs) were then constrained within the mean GM mask. Third, BOLD signals in GM were averaged within each of the ROIs to produce mean time series, which were subsequently used to derive pairwise temporal correlations among GM regions. To improve the signal-to-noise ratio (SNR) in WM, BOLD signals were spatially smoothed within the mean WM mask using a 3-mm full-width half-maximum isotropic Gaussian kernel. Finally, all-time series in GM and WM were normalized to zero mean and unit variance, which were then interpolated from 200 into 400 time points using spline interpolations. The normalization of all-time series in GM and WM to zero mean and unit variance transforms GM and WM signals into comparable intensity ranges to facilitate convolution-based lag time computations, correlation analysis, and subsequent group averaging.

BOLD time series in 3 representative GM ROIs, namely the left PCC, left intraparietal sulcus (IPS), and right opercular part of inferior frontal gyrus (IFGoperc), were used as references in our analyses. The rationale for the selection of these ROIs is as follows: The PCC is a key node of the DMN, which is activated under resting conditions (Raichle et al. 2001) and whose time courses may be representative of task-negative networks. The IPS is a major component of the dorsal attention network negatively correlated with the DMN (Vossel et al. 2014), and is generally active during task conditions. A third GM ROI, the right IFGoperc, was also included because it was found to have minimum temporal correlations with the left PCC, which thus could serve as a “nuisance” GM region in a resting brain. Note that as the IPS is not parcellated in the AAL atlas, it was manually defined in this study as a 13-voxel cube centered at peak coordinates (MNI coordinates: −28, −58, 46) of IPS activation reported previously (Battista et al. 2018). Correlation coefficients (CCs) between the left PCC and left IPS and right IFGoperc are given in the black boxes in Supplementary Figure 1. Note that both the CCs were very small, indicating that BOLD signals in the left IPS and right IFGoperc were independent of those in the left PCC.

### Detection of HRF in GM and WM

**Figure 1:**
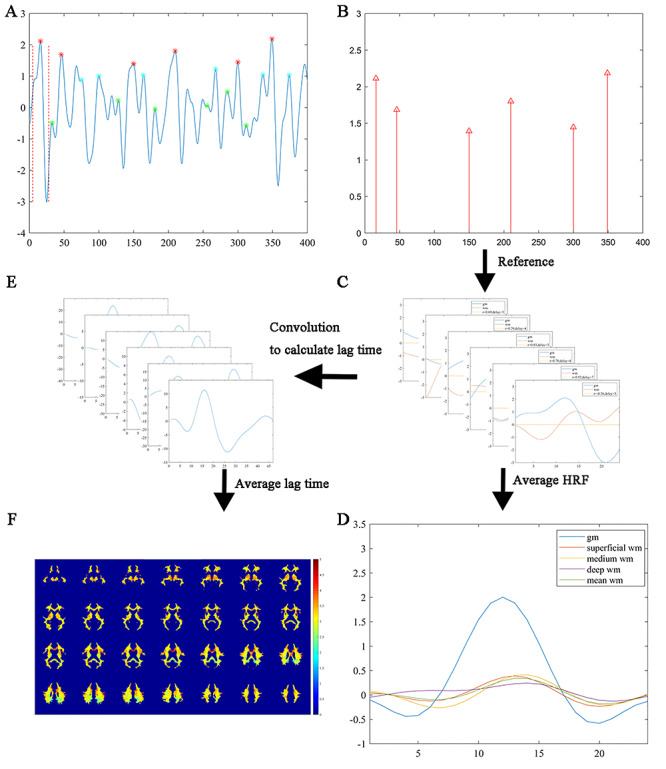
Schematic diagram of the proposed workflow. (*A*) An example of 3 groups of local signal maxima (6 for each group marked in red, cyan, and green asterisks, respectively) corresponding to local spontaneous neural activities at 3 amplitudes (high, medium, and low) were identified in a GM region. HRFs triggered by the neural activities are defined as 11 s before and 12 s after the local maxima. (*B*) Positions of the local maxima in the red group in (*A*). (*C*) Derivations of WM HRFs for individual neural activities by aligning the time window of each activity extracted in the GM region. (*D*) Average of 6 HRFs in GM and WM. Orange, yellow, and purple color denotes the HRF in representative voxels in superficial (voxel 1), medium (voxel 2), and deep (voxel 3) WM, respectively, and green color denotes the mean of the WM HRFs. (*E*) Six lag times are computed by finding local maxima of convolution between GM HRFs and BOLD signals in WM. (*F*) Color-coded average lag time superimposed onto the WM mask.

The BOLD HRF is a key feature in characterizing neural activities in fMRI, the derivation of which helps elucidate the relationship between GM and WM neural activities in this study. Figure 1 presents the workflow of the experimental procedures used in this work. First, 6 local peaks with high amplitude (denoted by red asterisks in Fig. 1*A*) were identified from the BOLD signal of each representative GM ROI. A time window of 24 s centered at each of the signal peaks was then used to extract spontaneous brain activities, termed neuronal activity avalanches (Liu and Duyn 2013), within which the voxel-averaged fMRI time course was obtained. Meanwhile, the HRF in each WM voxel was estimated by averaging the time series in the same time window as for the reference GM. Finally, an average HRF in each GM reference and WM voxel was calculated by averaging the 6 individual HRFs within each subject, and then by averaging across all the subjects.

In order to examine whether the derived WM HRFs vary with the amplitude of activity avalanches in reference GM, 3 groups of local peaks were defined at high, medium, and low levels of amplitude (denoted by the red, cyan, and green asterisks, respectively in Fig. 1*A*) in the BOLD time series in the PCC and were used separately to derive WM HRFs similarly as above. The quantitative standards of high, medium, and low levels are as follow: First of all, all local signal peaks (except those within 10 s of the 2 ends) were identified and sorted into a descending order of the amplitude. To select peaks of high level, the first 6 peaks were chosen as an initial candidate list, and their consecutive time differences were recorded. Any peak with a time difference of <5 s to the preceding one would be excluded and a new peak in the sorted peaks would then be recruited to the end of the list. This procedure was repeated until all 6 peaks had consecutive time differences of ≥5 s. To select medium and low levels of peaks, the first and last 6 peaks were chosen respectively from the remaining sorted peaks and iterative procedures as above were implemented. An example of high, medium, and low levels of peaks from one subject is shown in Figure 1*A*. For further comparisons, WM HRFs were also derived from HRFs in the left PCC defined using 6 and 100 random time points in the BOLD time series, respectively.

Furthermore, to validate the effectiveness of the proposed method, we compared HRFs extracted from WM signals with those obtained by shuffling the signal phases. Specifically, a fast discrete Fourier transform was applied to an original WM time series, and then an inverse Fourier transform was used to reconstruct a new WM time series after random phase-shuffling of the component frequencies while keeping their amplitudes the same. The time axis of the reconstructed signal was aligned to that of each avalanche extracted from the BOLD time series in the left PCC, and the WM HRF of the perturbed time series was similarly derived.

### Calculation of WM Lag Time Relative to GM References

For each of the 3 representative GM ROIs, individual GM HRFs corresponding to avalanches and corresponding derived WM HRFs were first convolved together. Lag time was then estimated from the location of the maximum value in the convolution product. To enhance the stability and precision of lag time computations, the average lag time was calculated across 6 reference avalanches and then smoothed with a kernel of 3 × 3 × 3 voxels for each subject as implemented in Mitra et al. (2015). The smoothed lag times were subsequently averaged across all subjects and mapped onto the mean WM mask.

In order to characterize the dependency of WM lag time on the distance of WM voxels to reference GM, WM masks with 3 different depths, namely superficial, medium, and deep, were defined. Specifically, the WM depth mask was created by successively dilating each GM reference 100 times using a square structuring element with size of 3 × 3 voxels and then multiplying with the mean WM mask. The intersection of WM mask and each GM reference dilated at 1st–10th, 11th–20th and >20th times were defined respectively as superficial, medium, and deep WM relative to the GM reference (see Supplementary Fig. 2). The WM stratified as such allowed a similar number of voxels in each of these layers. Finally, for each of the GM references, the resulting WM mask at 3 different depths was masked onto the WM lag time map to allow for computations of average WM lag time in each of the 3 WM depths.

### Correlation analysis between the derived WM HRFs and reference GM HRFs

To explore the relationship of the HRFs between WM and GM, pairwise Pearson’s correlations were sought between each HRF of the 3 GM references and corresponding derived WM HRFs for each subject. The maps of CCs were confined within the mean WM mask and averaged across all subjects, and differences between the correlation map with respect to each of the GM references and the mean of the 3 maps were computed.

### Statistical testing

Differences in HRF profiles between GM and WM as well as among different depths of WM were statistically analyzed. Specifically, for each reference GM region, differences in the mean lag time among the superficial, medium, and deep WM were first examined. Second, for each WM depth relative to the left PCC, the peak amplitude of WM HRFs derived from high, medium, and low amplitude of the reference GM HRF was compared. Third, distribution patterns of lag time in WM with respect to 3 different GM references were assessed. Lastly, differences in distribution patterns of the coefficient of correlation between derived WM HRFs and GM HRFs in 3 reference regions were explored. In all these analyses, paired 2 sample *t*-tests were used with significance level of *P* < 0.01.

### Probabilistic Fiber Tracking

DWIs were processed using the diffusion toolbox of Functional Magnetic Resonance Imaging of the Brain (FMRIB) Software Library, version 5 (Jenkinson et al. 2012). First, DWIs were corrected for head motion and image distortions (stretches and shears) due to eddy currents with affine transformation. Second, estimation of diffusion parameters by sampling crossing fiber models with a Bayesian framework was used to model diffusion signals as ball (isotropic) and stick (anisotropic) components, so as to generate a distribution of likely fiber orientations as well as an estimate of the uncertainty on these orientations within each voxel. Third, the 3 representative GM ROIs created in the fMRI analysis section, namely the left PCC, left IPS, and right IFGoperc, were used as seed regions to reconstruct ROI-to-voxel fiber tracts in the whole-brain WM. In order to coregister the GM ROIs from MNI space to the DWI space, (forward and backward) transformation matrices were created by first coregistering the B_0_ image to the T_1_ images in the native space and then normalizing them to the MNI space using the Linear Imager Registration Tool supplied in FMRIB library. The GM ROIs were then backward transformed from the MNI space to the native space of DWI to guide probabilistic tractography. Fourth, fiber tracking was initiated from all voxels in each ROI to generate 5000 streamline samples, with a step length of 0.5 mm, a curvature threshold of 0.2, and a maximum of 2000 steps. Connectivity distributions from the ROI were generated, in which each brain voxel had a value representing its connectivity to the seed mask. Subsequently, connectivity distributions for each subject were forward transformed to the MNI space and then binarized with a preset threshold (number of voxels in the seed mask × 5000 × 0.005). The final connectivity map was obtained by summing up individual binarized maps, which was thresholded at >75% of the subject account.

## Results

### Detections of Resting State HRFs in WM

**Figure 2:**
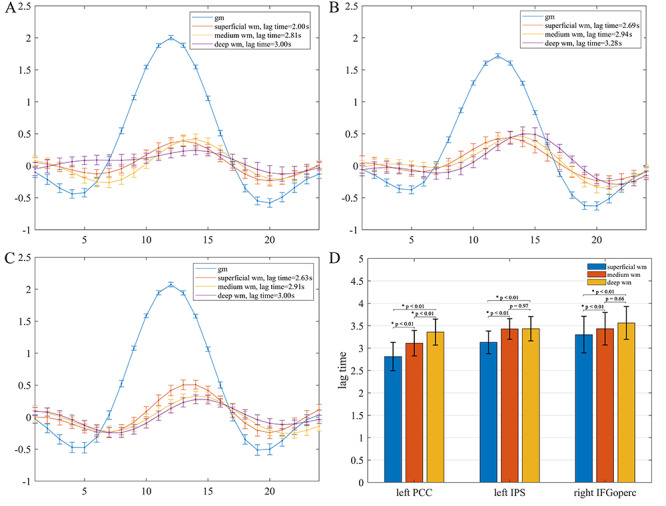
Average HRFs of GM regions and WM voxels and statistical comparisons. (*A–C*) HRFs of the left PCC, left IPS, and right IFGoperc and derived HRFs of WM at different distances to the GM regions. HRFs of GM regions are denoted in blue color, HRFs of representative voxels in superficial, medium, and deep WM are denoted in orange, yellow, and purple color, respectively. The error bars are the standard errors of the mean. (*D*) Mean and standard deviation of lag time of superficial, medium, and deep WM relative to the left PCC, left IPS, and right IFGoperc, respectively, and pairwise comparisons using 2 sample *t*-test.

HRFs derived for the left PCC, left IPS, and right IFGoperc are depicted in Figure 2*A–C*, in which blue color denotes the HRF in GM, and orange, yellow, and purple colors respectively denote the HRF in superficial, medium, and deep WM relative to the corresponding GM reference. The mean and standard deviation of WM lag time at each depth was 2.81 ± 0.32 s, 3.16 ± 0.29 s, and 3.35 ± 0.29 s for the left PCC reference, 3.13 ± 0.25 s, 3.43 ± 0.23 s, and 3.43 ± 0.27 s for left the IPS reference, and 3.30 ± 0.41 s, 3.48 ± 0.36 s, and 3.51 ± 0.37 s for the right IFGoperc reference, which are plotted in Figure 2*D*. It is quite apparent from Figure 2(*A–C*) that all WM HRFs manifested a similar shape with those of GM HRFs, but with a largely reduced peak amplitude and delayed peak time. Statistical analysis further revealed that the HRFs in WM varied with the relative distance to the GM regions (Fig. 2*D*). Specifically, the mean lag time became greater as the WM depth increased relative to the left PCC, with *P* < 0.01 for all 3 pairs of comparisons. For both the left IPS and right IFGoperc, the mean lag time in the superficial WM was smaller than in the medium and deep WM (*P* < 0.01), but there were no significant differences between the medium and deep WM. The trend of more delayed peak time in deeper WM is consistent with the report by Li et al. (2019), which supports the notion that WM signals are associated with activities in GM, but the response profiles depend on the relative distance to the GM cortex and distributions of local vasculature (Nonaka et al. 2003; Akashi et al. 2017).

**Figure 3:**
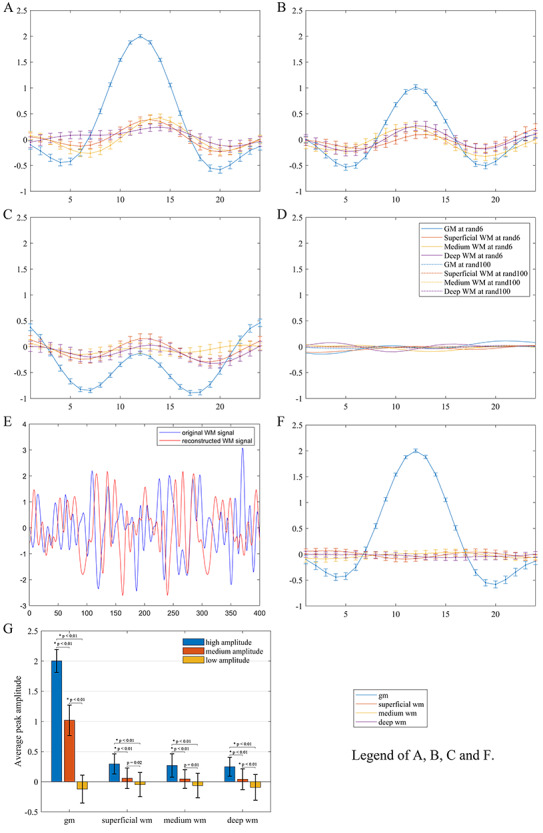
Derived average HRFs of WM voxels from different selections of BOLD time series in the left PCC. (*A–C*) Derived HRFs using neural activities at high, medium, and low amplitude, respectively. (*D*) Derived HRFs using neural activities at 6 and 100 random time points. (*E*) An example of BOLD time series in WM before (blue) and after (red) signal perturbation. The perturbation was implemented by randomly permuting the phases of the original time series in the Fourier spectrum but keeping the magnitudes intact. (*F*) Derived HRFs for 3 WM voxels at the same locations as in Figure 3(*A*) from BOLD time series perturbed as in (*E*) with the left PCC as the reference. (*G*) Paired *t*-tests of the peak amplitudes in the derived WM HRFs using neural activities at high, medium, and low amplitude of PCC. The error bars are the standard deviations.

The relationship of the derived WM HRFs and the reference GM HRFs in the left PCC at 3 different levels of BOLD signal amplitude (high, medium, and low) is shown in Figure 3(*A–C* and *G*). It can be observed that as the amplitude of PCC HRF decreased, the derived WM HRF became subdued and their characteristic profiles tended to diminish. The mean and standard deviation of the HRF amplitude in the left PCC at each peak amplitude were 2.00 ± 0.19, 1.02 ± 0.25, and − 0.12 ± 0.23, and of the derived WM HRFs were 0.30 ± 0.17, 0.06 ± 0.17, and − 0.05 ± 0.20, respectively in superficial WM, 0.27 ± 0.19, 0.05 ± 0.16, and − 0.06 ± 0.20, respectively in medium WM, and 0.25 ± 0.17, 0.04 ± 0.17, and − 0.09 ± 0.21, respectively in deep WM (see Fig. 3*G*). Statistical analysis further showed that the reference GM HRF in the left PCC had significant differences among the 3 peak amplitudes (*P* < 0.01). The derived mean peak amplitudes of WM HRFs at different depths all decreased as the peak amplitude of the reference GM HRF decreased. Specifically, in each WM depth, the peak amplitude of the WM HRF derived from high amplitude of GM HRF was higher than that derived from medium and low amplitude of GM HRF (*P* < 0.01). The decline trend did not reach the significance level of *P* < 0.01 at the superficial and medium WM depths when comparing the peak amplitude of WM HRFs derived from medium with that from low amplitude of reference GM HRFs. In particular, when the reference HRF was randomly sampled from 6 or 100 time points in the left PCC, virtually no HRFs could be derived from the WM (Fig. 3*D*).

To explore whether the observed coupling of HRFs in GM and WM was incurred by coincidences or methodological biases, a WM BOLD time series was reconstructed with randomly perturbed phases but intact magnitudes and used for HRF estimations by referencing the left PCC. The original and reconstructed BOLD time series are shown in Figure 3*E* (denoted as blue and red curves, respectively) and the derived WM HRF is given in Figure 3*F*. As expected, the derived WM HRF had near zero amplitude and no similarity with the reference GM HRF. This phenomenon, along with the amplitude dependency of WM responses shown above, essentially ruled out the possibility that the coupling of HRFs in GM and WM was coincidental or artificial.

### Distributions of WM Lag Time Relative to GM References

**Figure 4:**
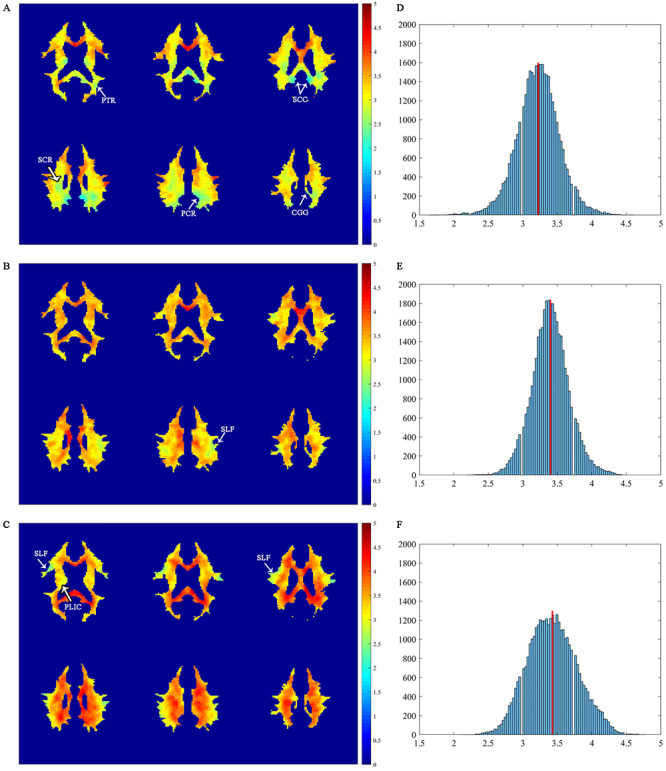
Distributions of WM lag time relative to the left PCC, left IPS, and right IFGoperc. (*A–C*) Maps of WM lag time superimposed onto the WM mask for the left PCC, left IPS, and right IFGoperc reference regions, respectively. Identifiable WM tracts with low lag time are annotated. (*D–F*) Histograms of WM lag time relative to the left PCC, left IPS, and right IFGoperc, respectively. The red line denotes the mean value of each histogram.

Maps of WM lag times relative to the left PCC, left IPS, and right IFGoperc are shown in Figure 4(*A–C*), with warmer colors denoting longer lag times. The lag time relative to the left PCC in (*A*), appeared to be generally shorter than that relative to the other 2 GM references in (*B,C*), with longest lag time relative to the right IFGoperc (*C*). The dependency of WM lag times on the associated GM regions suggests that the signal changes evoked within WM are produced by a mixture of neural activities associated with different GM regions.

Distributions of the WM lag time are summarized in histograms for each of the 3 GM references in the right column of Figure 4. All the histograms were nearly normally distributed, with mean values around 3.22, 3.40, and 3.43 s relative to the left PCC, left IPS and right IFGoperc, respectively (denoted as red lines in Fig. 4(*D–F*). Spatially, WM voxels with relatively short lag time tended to form clusters that resembled some of the known WM tracts. Specifically, in reference to the left PCC (Fig. 4*A*), identifiable WM tracts with short lag time included the cingulum (cingulate gyrus, CGG), splenium of corpus callosum (SCC), superior and posterior corona radiata (SCR, PCR), posterior thalamic radiation (PTR, including OR), all of which are close to the PCC. In reference to the IPS (Fig. 4*B*), WM tracts with short lag time included mainly the superior longitudinal fasciculus (SLF), and in reference to the IFGoprec (Fig. 4*C*), identifiable WM tracts with short lag time included the posterior limb of internal capsule (PLIC) and SLF. Differences in the WM lag time among the 3 GM references are shown in Supplementary Figure 3. It can be seen that, when compared to the WM lag times with both the left IPS (*A*) and right IFGoperc (*B*) as references, a large portion of WM had a significantly shorter lag time with the left PCC as the reference (*P* < 0.01). This portion of WM was mostly distributed near the posterior cortex of the brain (see the regions with negative T-statistics in Supplementary Fig. 3*A*, *B*). This indicates that the WM lag time depended both on GM reference and WM depth relative it, which may be related to hemodynamic variations in WM and the proximity of relevant vasculature to the WM site. When compared to the WM lag time with the right IFGoperc as the reference (*C*), a small portion of WM had significantly shorter lag time with the left IPS as the reference (*P* < 0.01). Detailed lag time distributions from individual subjects are provided in Supplementary Figure 4. It can be observed that, likely due to much weaker BOLD signals in WM, there is a fair amount of variabilities among the individual subjects. Our further analysis indicated that stable lag time distributions could be reached by averaging data from 12 subjects, which had high similarity with that computed from all the subjects studied, with a CC > 0.7 for all the 3 GM references.

### Resting State Correlations between WM and GM References

**Figure 5:**
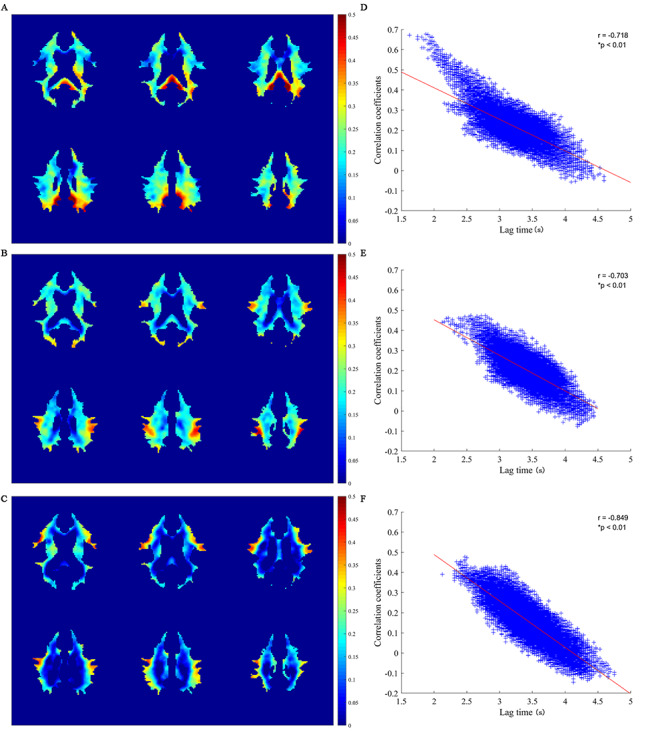
Maps of correlation coefficients between WM and GM and their relation with lag times. (*A–C*) Maps of correlation coefficient between the WM and the left PCC, left IPS, and right IFGoperc, respectively. (*D–F*) Scatter plots of WM lag time versus correlation coefficient relative to the left PCC, left IPS, and right IFGoperc, respectively. Each plus sign (+) represents 1 WM voxel.

Figure 5 shows maps of Pearson’s CCs in HRFs between each WM voxel and the 3 reference GM regions. As seen in the left column, different GM references corresponded to different patterns of WM correlations. The left PCC corresponded to most widespread correlations, and the IFGoperc corresponded to the least correlations. Close inspections of the WM voxels with high CCs with the reference GM regions (please see the red regions in the left column of Fig. 5) reveal correlation distributions tended to be oriented toward the locations of the reference regions, a phenomenon that was more pronounced for the left PCC and right IFGoperc. WM CCs with the 3 reference GM regions are compared in Supplementary Figure 5. It can be seen that a large portion of WM had greater correlations with the left PCC than with the left IPS (*A*) and right IFGoperc (*B*), with the most pronounced differences spatially close to the left PCC. It can also be appreciated that WM had relatively greater correlations with the left IPS than with the right IFGoperc in many WM regions (*C*). The correlation distributions were consistent with the WM lag time distributions in that the closer the WM region was to the GM reference, the smaller the lag time in WM, and the greater the correlation (Supplementary Fig. 5). This relation could be better revealed by further liner regression analysis between the lag times and CCs of WM relative to the 3 GM references (see the right column of Fig. 5), which exhibits strong negative correlations between them, with a regression CC of −0.72, −0.70 and −0.85 for the left PCC, left IPS, and right IFGoperc, respectively (*P* < 0.01).

### Probabilistic Fiber Tracking

Maps of connectivity to the left PCC are shown in Supplementary Figure 6. It can be seen that WM had widespread connections to the left PCC with high probability. In contrast, there were no structural connections to the left IPS and right IFGoperc at the same threshold level as for the left PCC (data not shown). The WM tracts connecting to the left PCC were primarily located in the CGG, SCC, PCR, and PTR, structures that are close to the left PCC. These tracts grossly overlapped with the structures with small lag time relative to the left PCC as shown in Figure 4(*A*) and Supplementary Figure 3(*A,B*). The consistency between the distribution of structural connectivity and the map of lag times supports that latencies of BOLD signals in WM are related to the structural connectivity responsible for transferring neural activity between cortical regions.

## Discussion

### General Findings

We have explored in this study the HRFs of BOLD effects in WM measured during a resting state. It was demonstrated that HRFs in WM can be derived by reference to avalanches of neural activity in GM, and these were found to exhibit reduced peak amplitudes and delayed peak times compared with those in GM. Distributions of lag times in WM depend on the functionality of GM references, and the amplitudes of derived WM HRFs are coupled with those of the GM HRF referenced. Specifically, the amplitudes of derived WM HRFs are proportional to those of the GM HRFs referenced, and diminish when HRFs in the reference GM are randomly chosen. In addition, analyses of correlations between WM and 3 representative GM regions reveal that the correlation patterns correspond to the functionality of the GM regions. Finally, distributions of WM tracts from probabilistic fiber tracking for each reference GM showed strong consistencies with the maps of WM lag time. These results converge to suggest that the WM BOLD signals are associated with the GM BOLD signals that encode changes in neural activities.

### Characteristics of WM HRFs

This study demonstrates that WM HRFs are derivable from GM HRFs by referencing activity avalanches in GM, and for the first time derives the temporal filtering that modulates BOLD signals in a resting state without requiring explicit onset times of neural events. Profiles of the derived WM HRFs include reduced peak amplitudes and delayed peak times as compared with GM HRFs, in agreement with our recent findings using event-related (Stroop color-word interference) task fMRI (Li et al. 2019). WM has much less dense vasculature than GM (Nonaka et al. 2003), with blood flow approximately one-fourth of the latter (Raichle et al. 2001), and thus the amplitudes of hemodynamic responses in WM are expected to be reduced. The delayed time to peak of WM HRFs has been well characterized in several reports from both resting state and task-related fMRI studies (Tae et al. 2014; Thomas et al. 2014; Erdoğan et al. 2016; Tong et al. 2017; Ding et al. 2018; Courtemanche et al. 2018). Additionally, the derived WM HRFs appeared to also have prolonged initial dips, a phenomenon that has been reported in several previous task-based studies (Menon et al. 1995; Yacoub et al. 2001; Hu and Yacoub 2012). HRFs in GM have been shown in some measurements to consist of a period of early initial dip, presumably originating from a rise in deoxyhemoglobin due to an increase in metabolism before subsequent blood flow increases (Hu and Yacoub 2012). If increased activity in GM causes an increase in energy demand in WM, then the prolonged initial dip in WM may be attributed to a longer arrival time of cerebral blood flow from feeding arteries in the pial matter than GM (Nonaka et al. 2003; Brown and Thore 2011; Giezendanner et al. 2016). In spite of the consistency with previous studies and vascular physiology, the initial dips we observed may contain undershoot, partly or even fully, from the preceding event, which we are unable to resolve in this work.

### Relations of Lag Time and BOLD Signal Correlations in WM

In this study, distributions of lag time in WM were found to be inversely related to maps of BOLD signal correlations with reference GM, i.e., WM regions with smaller latencies tend to have higher correlations with the GM. A shorter lag time may correspond to a greater degree of signal synchrony between WM and GM. It should be noted that the WM regions with small lag times are mostly located near the reference GM. Although WM regions more distant from the reference GM tend to have larger time delays and thus reduced signal correlations, BOLD signals in the distant WM regions are also more likely to be mixed with signals from other GM regions, which may further weaken the correlations.

### Structural Basis of WM Clusters

It was found that the WM voxels with small latencies and high correlations tend to cluster into distinct WM tracts such as CGG, SCC, SCR, PCR, and PTR, most of which have known structural connections with the PCC (Mamah et al. 2010). These structures were largely confirmed by our fiber tracking analyses. Anatomically, the PCC is connected with ventromedial prefrontal cortex and retrosplenial cortex, which are also parts of DMN (Kaboodvand et al. 2018), through the CGG (Heilbronner and Haber 2014) and dorsal SCC (Knyazeva 2013), respectively. The PCC receives a significant portion of the afferent axons from the superficial nucleus of the thalamus through the thalamocortical fibers as well (Leech and Sharp 2014), the latter of which are involved in the control of cortical arousal and consciousness (Xie et al. 2011). Although direct connections between the PCC and SCR or PCR have not been well documented, it has been reported that mean diffusivities are simultaneously increased in the PCC and SCR/PCR in patients with mild cognitive impairment (Thillainadesan et al. 2012) and Alzheimer’s disease (Li et al. 2016). It is worth noting that both the left IPS and right IFGoperc showed small latencies in SLF across different axial slices. SLF is an association fiber tract that structurally connects the IFG and inferior parietal lobule (Briggs et al. 2019). IFGoperc also showed small latency in the PLIC which contains 2-way tracts for transmission of information to and from all areas of the cerebral cortex, especially the primary motor and premotor cortex as well as sensory area (Lemaire et al. 2011). This is consistent with a report that the right IFGoperc cooperates with the premotor cortex and plays a role in inhibition and generation of a motor response (Hampshire et al. 2010).

### Methodological Considerations

Estimations of HRFs from resting state fMRI signals have been previously pursued by Tagliazucchi et al. (2012), which was subsequently refined by Wu et al. (2013), who innovated a more sophisticated blind deconvolution approach for this purpose. In both of these works, fMRI signal peaks with relatively large amplitudes are considered to reflect intrinsic spontaneous “events”, often referred to as brain activity avalanches (Beggs and Plenz 2003; Liu and Duyn 2013), which convolve with the tissue hemodynamic responses to produce the measured BOLD signals. It has been demonstrated that the spatiotemporal dynamics of fMRI time series can be efficiently captured by a small set of such intrinsic events (Tagliazucchi et al. 2012; Liu and Duyn 2013), the underpinning for which is long range correlations of avalanche activities across functionally connected brain regions (Cifre et al. 2017). Although these pioneering studies have established the validity and possibility of extracting GM HRFs from resting state fMRI signals on the basis of brain activity avalanches, extensions to derivations of WM HRFs need further investigations. As mentioned previously, we have recently found in an event-related study that BOLD signals in WM under functional tasks exhibit response profiles quite distinct from those in GM, which tends to suggest that WM HRFs under resting conditions might also differ. Therefore, we began our explorations of resting state HRFs in WM by employing more basic methods as proposed in Tagliazucchi et al. (2012), such that potential confounds that advanced methods may entail could be avoided. Furthermore, we use detected avalanches in GM rather than in WM as references. This is based on our assumption that WM activities are associated with reference GM and consideration that WM BOLD signals are much weaker than GM (Huang et al. 2018), thus providing an unreliable basis for establishing HRF references. As a side note, the relation of BOLD signals in GM and WM has been recently studied by Tarun et al. (2020) with an elegant graph-based mathematical framework. The goal of this work, however, is to interpolate BOLD signals in WM rather than detecting HRFs from measured WM signals, as is the goal in our work.

### Alternative Interpretations

With respect to interpretations of fluctuations in resting state WM fMRI signals, a study by Özbay et al. (2018) reported that brain fMRI signals are in part mediated by extrinsic sympathetic activities, which in principle can induce instantaneous signal fluctuations in WM. However, the observed sympathetic activities in 8 carefully chosen participants they studied had variable frequencies with typical range of 0–0.03 Hz, and thus contribute mostly to low frequency components of spontaneous brain activities. Moreover, the impact of sympathetic activities on fMRI signals was found to be pronounced only when participants were in moderate sleep. Therefore, although sympathetic activities may contribute to some of the variations in fMRI signals, the effects should not dominate in a waking resting state study, particularly in light of our observations that the distribution of signal latencies and correlation profiles in WM are relative to the GM reference chosen (which rules out other system level effects as well). The signal latencies observed in WM, on the other hand, might well be interpreted as draining vein effects from cortical GM. However, as pointed out in our earlier work (Huang et al. 2018; Li et al. 2019), there are 2 distinct blood draining subsystems in the brain that serve WM and cortical GM separately. Specifically, the first subsystem is a superficial system that drains the cerebral cortex and subcortical WM (i.e. the outer centimeter of superficial WM), whose venous drainage collects deoxygenated blood into the pial veins located at the surface of the brain. The second subsystem is a deep system that drains deep WM tracts via the subependymal veins, which are close to the lateral ventricles (San Millán Ruíz et al. 2009). As such, there are no vascular interactions between the 2 spatial domains, and under normal physiology, the blood flow out of activated GM and superficial WM is unable to reach deep WM to modulate the signals therein (Sarwar and McCormick 1978). Finally, we should note that the underlying mechanism for our derivations of WM HRFs is “matched filtering”. One may speculate that the derived HRFs might be simply from random noises, as could occur due to the inherent property of this type of operations. Our experiments, however, demonstrate that distribution patterns of the derived HRFs depended on the relative location of WM to GM. Moreover, when BOLD signals in WM were randomly phase-shuffled, virtually no HRFs could be derived (see Fig. 3*E*,*F*), which basically excluded the possibility that the WM HRFs we derived were random noise artifacts.

### Limitations

It should be pointed out that our derivations of WM HRFs by nature are data driven. Certainly the data driven analysis is unable to determine the timing of avalanche activities precisely. Ideally, using measurements from independent techniques such as electroencephalograms or local field potentials could provide more precise localizations of spontaneous neural events. However, such approaches are still far from optimal and thus are not widely applicable yet (Wu et al. 2013). In addition, there is a common concern over studies of WM BOLD signals that whether the detected signals are due to residual partial volume effects from adjacent GM. In the present study, precautions were taken to minimize the potential effects of partial volume averaging from GM signals. First, the WM mask, which was obtained by segmenting the T_1_ weighted images of each individual subject to confine the spatial domain of WM signals, was given a tight threshold of >80%. This could remove the subcortical transition zone between GM and superficial WM. Second, the WM HRFs were derived on the basis of voxel-wise analyses, which further reduced the possibility of signal contaminations from GM nearby. Meanwhile, fMRI data may contain confounding effects from cardiopulmonary activities, subjects’ head movements, global sympathetic tones, and other artifacts (Power et al. 2017) that are particularly pronounced in WM. To ameliorate this problem, we did not extract the WM HRFs by directly finding local signal maxima from the WM time series as we did for the GM HRFs since neural activities at local signal maxima might originate from artifacts. GM BOLD signals, on the other hand, typically have much higher SNR ratio and thus can be used as a more reliable basis for identifying intrinsic neural activities, which were extracted for references in this study. To further suppress the impact of spurious signal peaks in WM, our WM HRFs were derived by averaging the estimates made with 6 GM HRFs at different local maxima of the time series. Lastly but not least importantly, the temporal resolution of 2 s used in this study is somehow limited. We recognize this limitation and thus employ spline interpolations to increase the nominal resolution of the signals (Mitra et al. 2015). Ultimately, the accuracy of lag time calculations will benefit from using higher temporal resolutions such as “multiband” or “simultaneous multi-slice” (SMS) acquisitions protocols. However, the benefit of improved temporal resolutions and the cost of degraded image quality, which often comes with rapid imaging, should be traded-off optimally before these protocols become widely adopted (Demetriou et al. 2018). In the near future, we will systemically evaluate the performance of the SMS technology, and employ an optimized protocol in our functional studies.

## Conclusion

This study investigates HRFs of BOLD effects in WM from resting state fMRI. It demonstrates that resting state HRFs in WM can be derived by referencing to activity avalanches in GM. The derived resting state WM HRFs tend to have lower peak amplitudes and delayed peak times compared with those in GM. Distributions of lag times and temporal correlations in WM depend on the GM reference chosen. These findings suggest that BOLD signals in WM may encode neural activities associated with those in GM.

## Notes

*Conflict of interest:* None declared 

## Funding

National Institutes of Health [R01 NS093669 and R01 NS113832 to J.C.G.] and the National Natural Science Foundation [grant number 61806029]; the Chengdu University of Information Engineering Research Fund [grant number KYTZ201719]; and the Research Fund from Department of Education of Sichuan Province [grant numbers 18ZA0089].

## Supplementary Material

ccc-Supplementary-Information_tgaa056

## References

[ref1] Akashi T, Takahashi S, Mugikura S, Sato S, Murata T, Umetsu A, Takase K. 2017. Ischemic white matter lesions associated with medullary arteries: classification of MRI findings based on the anatomic arterial distributions. American Journal of Roentgenology 209:W160–W168. 28678575 10.2214/AJR.16.17231

[ref2] Battista C, Evans TM, Ngoon TJ, Chen T, Chen L, Kochalka J, Menon V. 2018. Mechanisms of interactive specialization and emergence of functional brain circuits supporting cognitive development in children. NPJ Science of Learning. 3:1–11. 30631462 10.1038/s41539-017-0017-2 PMC6220196

[ref3] Beggs JM, Plenz D. 2003. Neuronal avalanches in neocortical circuits. The Journal of Neuroscience 23:11167–11177. 14657176 10.1523/JNEUROSCI.23-35-11167.2003 PMC6741045

[ref4] Behzadi Y, Restom K, Liau J, Liu TT. 2007. A component based noise correction method (CompCor) for BOLD and perfusion based fMRI. NeuroImage 37:90–101. 17560126 10.1016/j.neuroimage.2007.04.042 PMC2214855

[ref5] Biswal B, Zerrin Yetkin F, Haughton VM, Hyde JS. 1995. Functional connectivity in the motor cortex of resting human brain using echo-planar MRI. Magnetic Resonance in Medicine 34:537–541. 8524021 10.1002/mrm.1910340409

[ref6] Briggs RG, Chakraborty AR, Anderson CD, Abraham CJ, Palejwala AH, Conner AK, Pelargos PE, O'Donoghue DL, Glenn CA, Sughrue ME. 2019. Anatomy and white matter connections of the inferior frontal gyrus. Clinical Anatomy 32:546–556. 30719769 10.1002/ca.23349

[ref7] Brown WR, Thore CR. 2011. Cerebral microvascular pathology in ageing and neurodegeneration. Neuropathology and Applied Neurobiology 37:56–74. 20946471 10.1111/j.1365-2990.2010.01139.x PMC3020267

[ref8] Cifre I, Zarepour M, Horovitz SG, Cannas S, Chialvo DR. 2017. On why a few points suffice to describe spatiotemporal large-scale brain dynamics. arXiv preprint arXiv:1707.00759.

[ref9] Courtemanche MJ, Sparrey CJ, Song X, MacKay A, D'Arcy RC. 2018. Detecting white matter activity using conventional 3 Tesla fMRI: an evaluation of standard field strength and hemodynamic response function. NeuroImage 169:145–150. 29229580 10.1016/j.neuroimage.2017.12.008

[ref10] Demetriou L, Kowalczyk OS, Tyson G, Bello T, Newbould RD, Wall MB. 2018. A comprehensive evaluation of increasing temporal resolution with multiband-accelerated protocols and effects on statistical outcome measures in fMRI. NeuroImage 176:404–416. 29738911 10.1016/j.neuroimage.2018.05.011

[ref11] Ding Z, Huang Y, Bailey SK, Gao Y, Cutting LE, Rogers BP, Newton AT, Gore JC. 2018. Detection of synchronous brain activity in white matter tracts at rest and under functional loading. Proceedings of the National Academy of Sciences 115:595–600. 10.1073/pnas.1711567115 PMC5776967 29282320

[ref12] Ding Z, Newton AT, Xu R, Anderson AW, Morgan VL, Gore JC. 2013. Spatio-temporal correlation tensors reveal functional structure in human brain. PLoS One 8:e82107. 24339997 10.1371/journal.pone.0082107 PMC3855380

[ref13] Erdoğan SB, Tong Y, Hocke LM, Lindsey KP, deB Frederick B. 2016. Correcting for blood arrival time in global mean regression enhances functional connectivity analysis of resting state fMRI-BOLD signals. Frontiers in Human Neuroscience 10:311. 27445751 10.3389/fnhum.2016.00311 PMC4923135

[ref14] Fraser LM, Stevens MT, Beyea SD, D’Arcy RC. 2012. White versus gray matter: fMRI hemodynamic responses show similar characteristics, but differ in peak amplitude. BMC Neuroscience 13:91. 22852798 10.1186/1471-2202-13-91 PMC3469381

[ref15] Giezendanner S, Fisler MS, Soravia LM, Andreotti J, Walther S, Wiest R, Dierks T, Federspiel A. 2016. Microstructure and cerebral blood flow within white matter of the human brain: a TBSS analysis. PLoS One 11:e0150657. 26942763 10.1371/journal.pone.0150657 PMC4778945

[ref16] Grajauskas LA, Frizzell T, Song X, D'Arcy RC. 2019. White matter fMRI activation cannot be treated as a nuisance regressor: overcoming a historical blind spot. Frontiers in Neuroscience 13:1024. 31636527 10.3389/fnins.2019.01024 PMC6787144

[ref17] Hampshire A, Chamberlain SR, Monti MM, Duncan J, Owen AM. 2010. The role of the right inferior frontal gyrus: inhibition and attentional control. NeuroImage 50:1313–1319. 20056157 10.1016/j.neuroimage.2009.12.109 PMC2845804

[ref18] Heilbronner SR, Haber SN. 2014. Frontal cortical and subcortical projections provide a basis for segmenting the cingulum bundle: implications for neuroimaging and psychiatric disorders. The Journal of Neuroscience 34:10041–10054. 25057206 10.1523/JNEUROSCI.5459-13.2014 PMC4107396

[ref19] Hu X, Yacoub E. 2012. The story of the initial dip in fMRI. NeuroImage 62:1103–1108. 22426348 10.1016/j.neuroimage.2012.03.005 PMC3389272

[ref20] Huang Y, Bailey SK, Wang P, Cutting LE, Gore JC, Ding Z. 2018. Voxel-wise detection of functional networks in white matter. NeuroImage 183:544–552. 30144573 10.1016/j.neuroimage.2018.08.049 PMC6226032

[ref21] Jenkinson M, Beckmann CF, Behrens TE, Woolrich MW, Smith SM. 2012. FSL. NeuroImage 62:782–790. 21979382 10.1016/j.neuroimage.2011.09.015

[ref22] Kaboodvand N, Bäckman L, Nyberg L, Salami A. 2018. The retrosplenial cortex: a memory gateway between the cortical default mode network and the medial temporal lobe. Human Brain Mapping 39:2020–2034. 29363256 10.1002/hbm.23983 PMC6866613

[ref23] Knyazeva MG. 2013. Splenium of corpus callosum: patterns of interhemispheric interaction in children and adults. Neural Plasticity 2013:639430. 23577273 10.1155/2013/639430 PMC3610378

[ref24] Leech R, Sharp DJ. 2014. The role of the posterior cingulate cortex in cognition and disease. Brain 137:12–32. 23869106 10.1093/brain/awt162 PMC3891440

[ref25] Lemaire J-J, Cosnard G, Sakka L, Nuti C, Gradkowski W, Mori S, Hermoye L. 2011. White matter anatomy of the human deep brain revisited with high resolution DTI fibre tracking. Neuro-Chirurgie 57:52–67. 21530985 10.1016/j.neuchi.2011.04.001

[ref26] Li M, Newton AT, Anderson AW, Ding Z, Gore JC. 2019. Characterization of the hemodynamic response function in white matter tracts for event-related fMRI. Nature Communications 10:1–11. 10.1038/s41467-019-09076-2 PMC6408456 30850610

[ref27] Li X, Wang H, Tian Y, Zhou S, Li X, Wang K, Yu Y. 2016. Impaired white matter connections of the limbic system networks associated with impaired emotional memory in Alzheimer's disease. Frontiers in Aging Neuroscience 8:250. 27833549 10.3389/fnagi.2016.00250 PMC5081353

[ref28] Liu X, Duyn JH. 2013. Time-varying functional network information extracted from brief instances of spontaneous brain activity. Proceedings of the National Academy of Sciences 110:4392–4397. 10.1073/pnas.1216856110 PMC3600481 23440216

[ref29] Mamah D, Conturo TE, Harms MP, Akbudak E, Wang L, McMichael AR, Gado MH, Barch DM, Csernansky JG. 2010. Anterior thalamic radiation integrity in schizophrenia: a diffusion-tensor imaging study. Psychiatry Research: Neuroimaging 183:144–150. 10.1016/j.pscychresns.2010.04.013 PMC3887223 20619618

[ref30] Marussich L, Lu K-H, Wen H, Liu Z. 2017. Mapping white-matter functional organization at rest and during naturalistic visual perception. NeuroImage 146:1128–1141. 27720819 10.1016/j.neuroimage.2016.10.005 PMC5321894

[ref31] Menon RS, Ogawa S, Hu X, Strupp JP, Anderson P, Uǧurbil K. 1995. BOLD based functional MRI at 4 Tesla includes a capillary bed contribution: echo-planar imaging correlates with previous optical imaging using intrinsic signals. Magnetic Resonance in Medicine 33:453–459. 7760717 10.1002/mrm.1910330323

[ref32] Mitra A, Snyder AZ, Blazey T, Raichle ME. 2015. Lag threads organize the brain’s intrinsic activity. Proceedings of the National Academy of Sciences 112:E2235–E2244. 10.1073/pnas.1503960112 PMC4418865 25825720

[ref33] Nonaka H, Akima M, Hatori T, Nagayama T, Zhang Z, Ihara F. 2003. Microvasculature of the human cerebral white matter: arteries of the deep white matter. Neuropathology 23:111–118. 12777099 10.1046/j.1440-1789.2003.00486.x

[ref34] Ogawa S, Lee T-M, Kay AR, Tank DW. 1990. Brain magnetic resonance imaging with contrast dependent on blood oxygenation. Proceedings of the National Academy of Sciences 87:9868–9872. 10.1073/pnas.87.24.9868 PMC55275 2124706

[ref35] Özbay PS, Chang C, Picchioni D, Mandelkow H, Moehlman TM, Chappel-Farley MG, van Gelderen P, de Zwart JA, Duyn JH. 2018. Contribution of systemic vascular effects to fMRI activity in white matter. NeuroImage 176:541–549. 29704614 10.1016/j.neuroimage.2018.04.045 PMC7328303

[ref36] Peer M, Nitzan M, Bick AS, Levin N, Arzy S. 2017. Evidence for functional networks within the human brain's white matter. The Journal of Neuroscience 37:6394–6407. 28546311 10.1523/JNEUROSCI.3872-16.2017 PMC6596606

[ref37] Power JD, Plitt M, Laumann TO, Martin A. 2017. Sources and implications of whole-brain fMRI signals in humans. NeuroImage 146:609–625. 27751941 10.1016/j.neuroimage.2016.09.038 PMC5321814

[ref38] Raichle ME. 2015. The brain's default mode network. Annual Review of Neuroscience 38:433–447. 10.1146/annurev-neuro-071013-014030 25938726

[ref39] Raichle ME, MacLeod AM, Snyder AZ, Powers WJ, Gusnard DA, Shulman GL. 2001. A default mode of brain function. Proceedings of the National Academy of Sciences 98:676–682. 10.1073/pnas.98.2.676 PMC14647 11209064

[ref40] Rostrup E, Law I, Blinkenberg M, Larsson H, Born AP, Holm S, Paulson O. 2000. Regional differences in the CBF and BOLD responses to hypercapnia: a combined PET and fMRI study. NeuroImage 11:87–97. 10679182 10.1006/nimg.1999.0526

[ref41] San Millán Ruíz D, Yilmaz H, Gailloud P. 2009. Cerebral developmental venous anomalies: current concepts. Annals of Neurology: Official Journal of the American Neurological Association and the Child Neurology Society. 66:271–283. 10.1002/ana.21754 19798638

[ref42] Sarwar M, McCormick WF. 1978. Intracerebral venous angioma: case report and review. Archives of Neurology 35:323–325. 646686 10.1001/archneur.1978.00500290069012

[ref43] Tae WS, Yakunina N, Kim TS, Kim SS, Nam E-C. 2014. Activation of auditory white matter tracts as revealed by functional magnetic resonance imaging. Neuroradiology 56:597–605. 24736936 10.1007/s00234-014-1362-y

[ref44] Tagliazucchi E, Balenzuela P, Fraiman D, Chialvo DR. 2012. Criticality in large-scale brain fMRI dynamics unveiled by a novel point process analysis. Frontiers in Physiology 3:15. 22347863 10.3389/fphys.2012.00015 PMC3274757

[ref45] Tarun A, Behjat H, Bolton T, Abramian D, Van De Ville D. 2020. Structural mediation of human brain activity revealed by white-matter interpolation of fMRI. NeuroImage 213:116718. 32184188 10.1016/j.neuroimage.2020.116718

[ref46] Thillainadesan S, Wen W, Zhuang L, Crawford J, Kochan N, Reppermund S, Slavin M, Trollor J, Brodaty H, Sachdev P. 2012. Changes in mild cognitive impairment and its subtypes as seen on diffusion tensor imaging. International Psychogeriatrics 24:1483–1493. 22452849 10.1017/S1041610212000270

[ref47] Thomas BP, Liu P, Park DC, Van Osch MJ, Lu H. 2014. Cerebrovascular reactivity in the brain white matter: magnitude, temporal characteristics, and age effects. Journal of Cerebral Blood Flow and Metabolism 34:242–247. 24192640 10.1038/jcbfm.2013.194 PMC3915204

[ref48] Tong Y, Lindsey KP, Hocke LM, Vitaliano G, Mintzopoulos D, Bd F. 2017. Perfusion information extracted from resting state functional magnetic resonance imaging. Journal of Cerebral Blood Flow and Metabolism 37:564–576. 26873885 10.1177/0271678X16631755 PMC5381451

[ref49] Tzourio-Mazoyer N, Landeau B, Papathanassiou D, Crivello F, Etard O, Delcroix N, Mazoyer B, Joliot M. 2002. Automated anatomical labeling of activations in SPM using a macroscopic anatomical parcellation of the MNI MRI single-subject brain. NeuroImage 15:273–289. 11771995 10.1006/nimg.2001.0978

[ref50] Vossel S, Geng JJ, Fink GR. 2014. Dorsal and ventral attention systems: distinct neural circuits but collaborative roles. The Neuroscientist. 20:150–159. 23835449 10.1177/1073858413494269 PMC4107817

[ref51] Wu G-R, Liao W, Stramaglia S, Ding J-R, Chen H, Marinazzo D. 2013. A blind deconvolution approach to recover effective connectivity brain networks from resting state fMRI data. Medical Image Analysis 17:365–374. 23422254 10.1016/j.media.2013.01.003

[ref52] Wu X, Yang Z, Bailey SK, Zhou J, Cutting LE, Gore JC, Ding Z. 2017. Functional connectivity and activity of white matter in somatosensory pathways under tactile stimulations. NeuroImage 152:371–380. 28284801 10.1016/j.neuroimage.2017.02.074 PMC5432381

[ref53] Xie G, Deschamps A, Backman S, Fiset P, Chartrand D, Dagher A, Plourde G. 2011. Critical involvement of the thalamus and precuneus during restoration of consciousness with physostigmine in humans during propofol anaesthesia: a positron emission tomography study. British Journal of Anaesthesia 106:548–557. 21285081 10.1093/bja/aeq415

[ref54] Yacoub E, Shmuel A, Pfeuffer J, Van De Moortele PF, Adriany G, Ugurbil K, Hu X. 2001. Investigation of the initial dip in fMRI at 7 Tesla. NMR in biomedicine: an international journal devoted to the development and application of magnetic resonance. In Vivo 14:408–412. 10.1002/nbm.715 11746933

[ref55] Yarkoni T, Barch DM, Gray JR, Conturo TE, Braver TS. 2009. BOLD correlates of trial-by-trial reaction time variability in gray and white matter: a multi-study fMRI analysis. PLoS One 4:e4257. 19165335 10.1371/journal.pone.0004257 PMC2622763

[ref56] Yeo BT, Krienen FM, Sepulcre J, Sabuncu MR, Lashkari D, Hollinshead M, Roffman JL, Smoller JW, Zöllei L, Polimeni JR. 2011. The organization of the human cerebral cortex estimated by intrinsic functional connectivity. Journal of Neurophysiology 106:1125. 21653723 10.1152/jn.00338.2011 PMC3174820

