# Supplementary material for: Hemodynamic Response Function in Brain White Matter in a Resting State
Source: Cereb Cortex Commun. 2020 Aug 28;1(1):tgaa056. doi: 10.1093/texcom/tgaa056 (PMC7552822; doi:10.1093/texcom/tgaa056)
Supplement: ccc-Supplementary-Information_tgaa056 [file ccc-supplementary-information_tgaa056.doc]

Supplementary Information for

Hemodynamic Response Function in Brain White Matter in a Resting State

Ting Wang, D. Mitchell Wilkes, Muwei Li, Xi Wu, John C. Gore, and Zhaohua Ding

Corresponding authors: Zhaohua Ding and Xi Wu

Email: zhaohua.ding@vanderbilt.edu or wuxi@cuit.edu.cn

**Supplementary Figures**


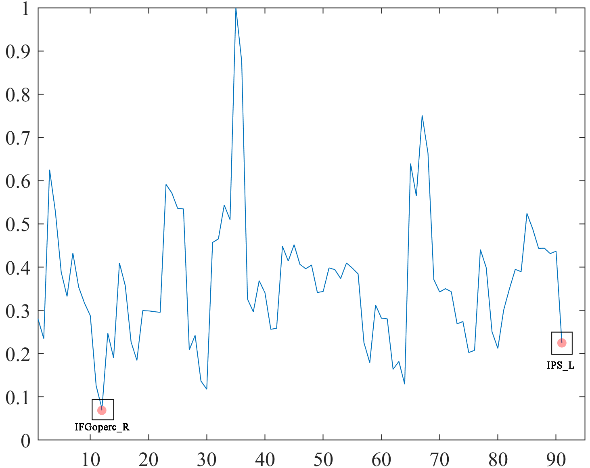


**Supplementary Fig. 1.** Correlation coefficients between the left PCC and other cortical regions. The horizontal axis denotes 91 cortical regions (90 from automatic anatomic labelling atlas plus the left IPS), and the vertical axis is the correlation coefficient between the left PCC and each cortical region. IPS_L and IFGoperc_R denotes the left IPS and right IFGoperc region respectively.


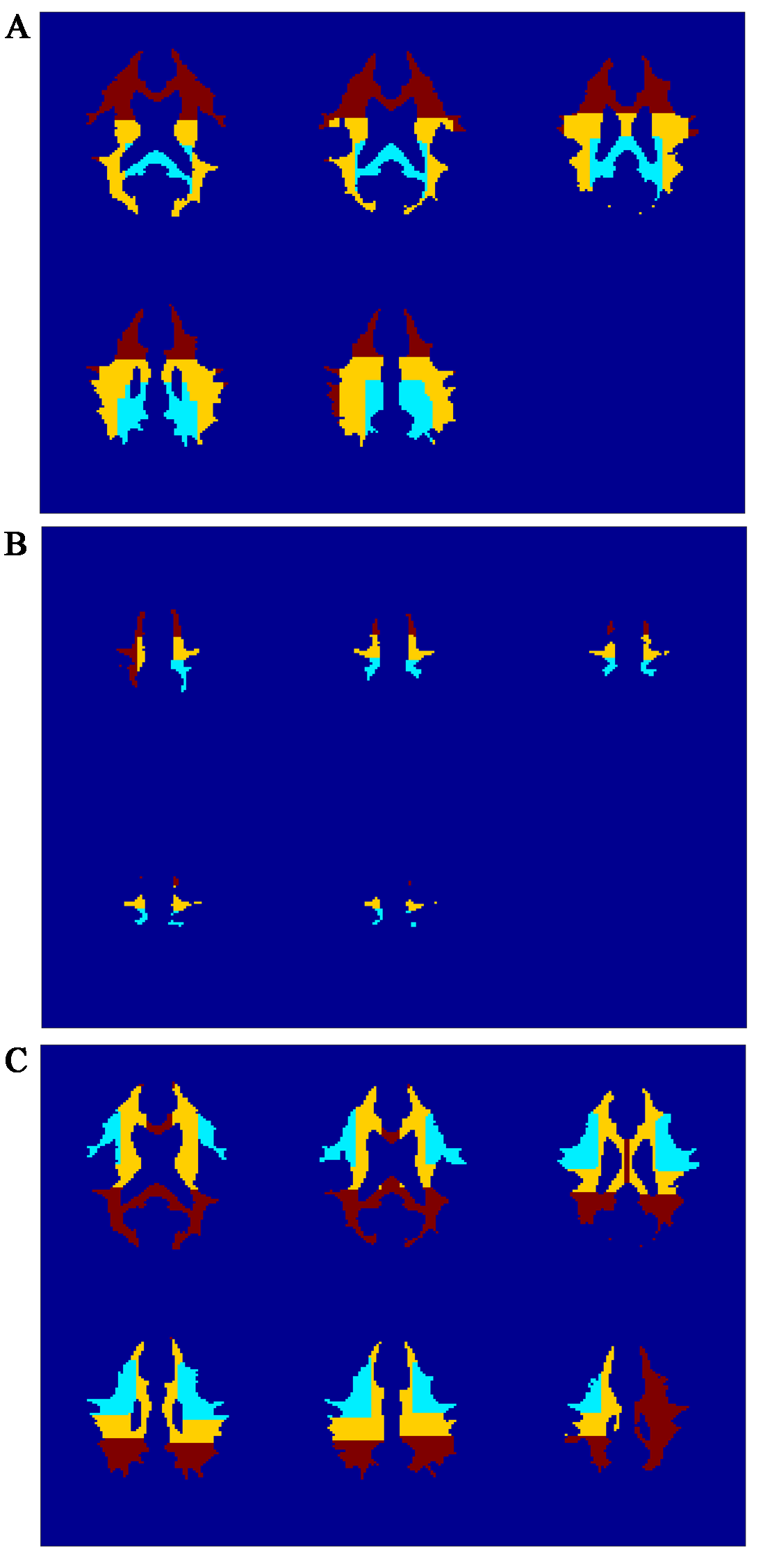


**Supplementary Fig. 2.** Superficial, medium and deep WM masks relative to bilateral PCC (A), IPS (B) and IFGoperc (C). Cyan, yellow and red colors denote the superficial, medium and deep WM corresponding to the referenced GM, respectively.


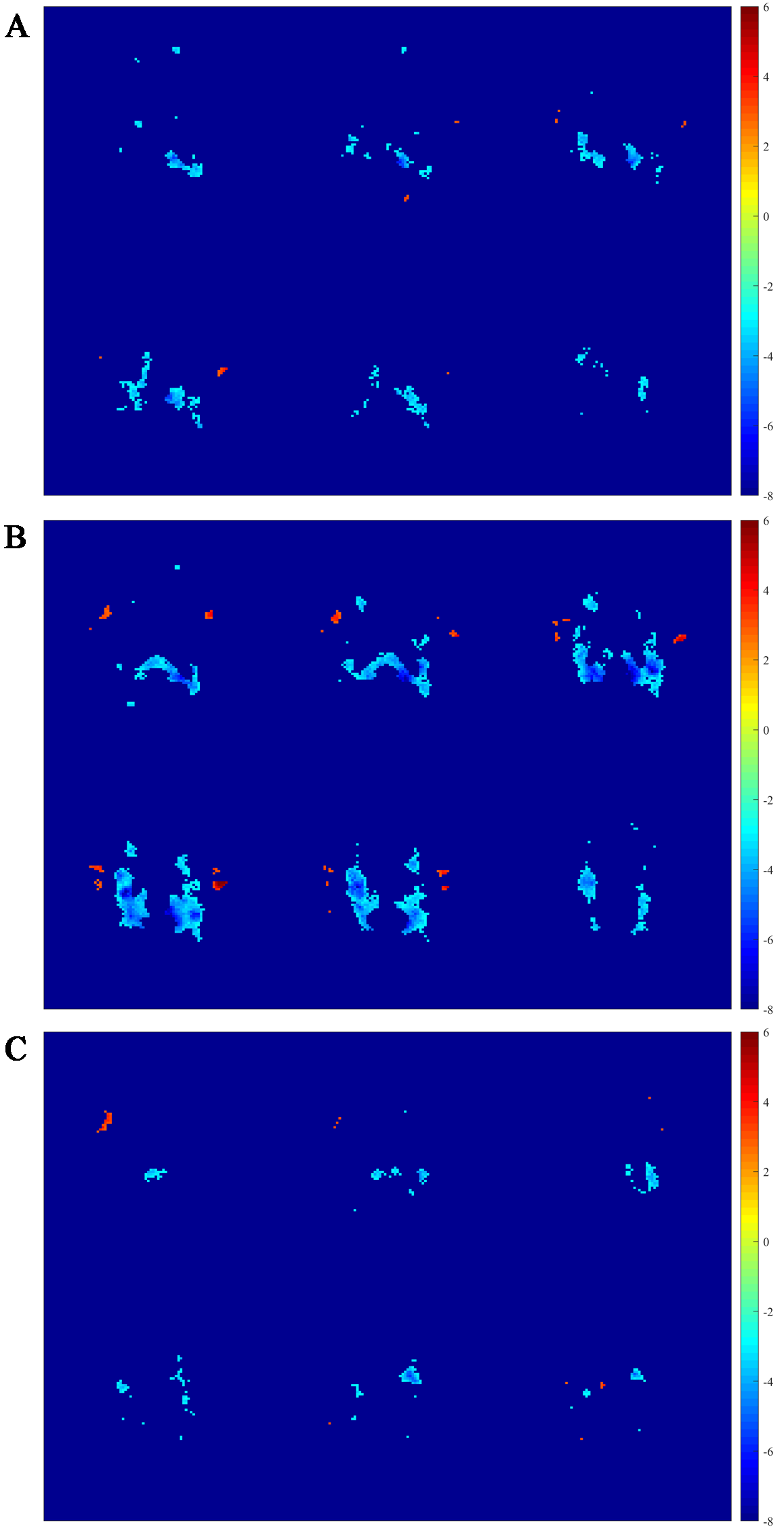


**Supplementary Fig. 3.** Maps of T-statistics of WM lag time among the three GM regions (paired t-test, p < 0.01, uncorrected). (A) Maps of T-statistic of WM lag time between the left PCC and the left IPS. (B) Maps of T-statistic of WM lag time between the left PCC and the right IFGoperc. (C) Maps of T-statistic of WM lag time between the left IPS and the right IFGoperc. The color bar denotes T-value. PTR, posterior thalamic radiation; SCC, splenium of corpus callosum; SCR, superior corona radiate; PCR, posterior corona radiate; CGG, cingulum (cingulate gyrus). PTR, posterior thalamic radiation; SCC, splenium of corpus callosum; SCR, superior corona radiate; PCR, posterior corona radiate; CGG, cingulum (cingulate gyrus).


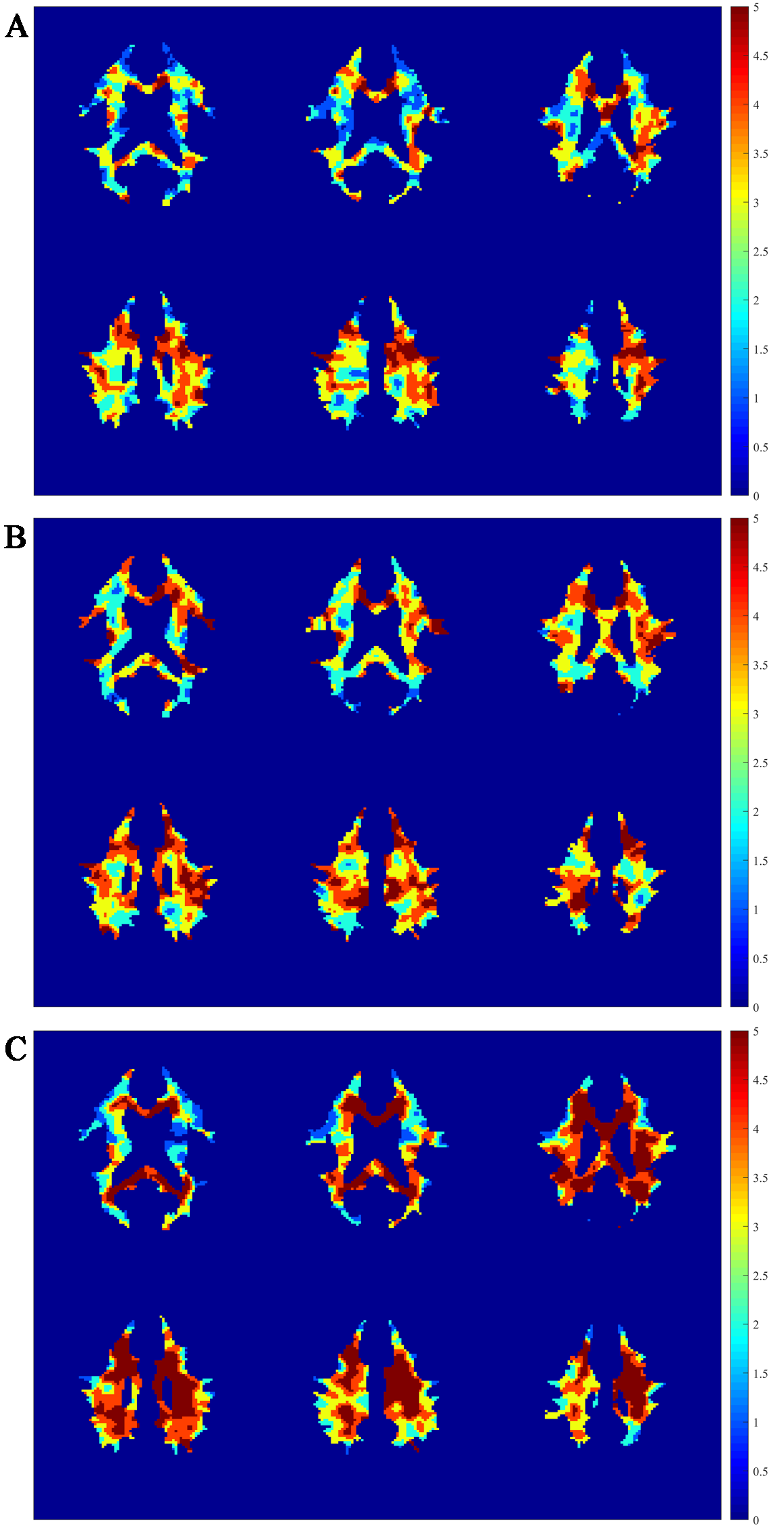


**Supplementary Fig. 4.** Distributions of WM lag time relative to the left PCC (A), the left IPS (B) and right IFGoperc (C) in 32 individual subjects.


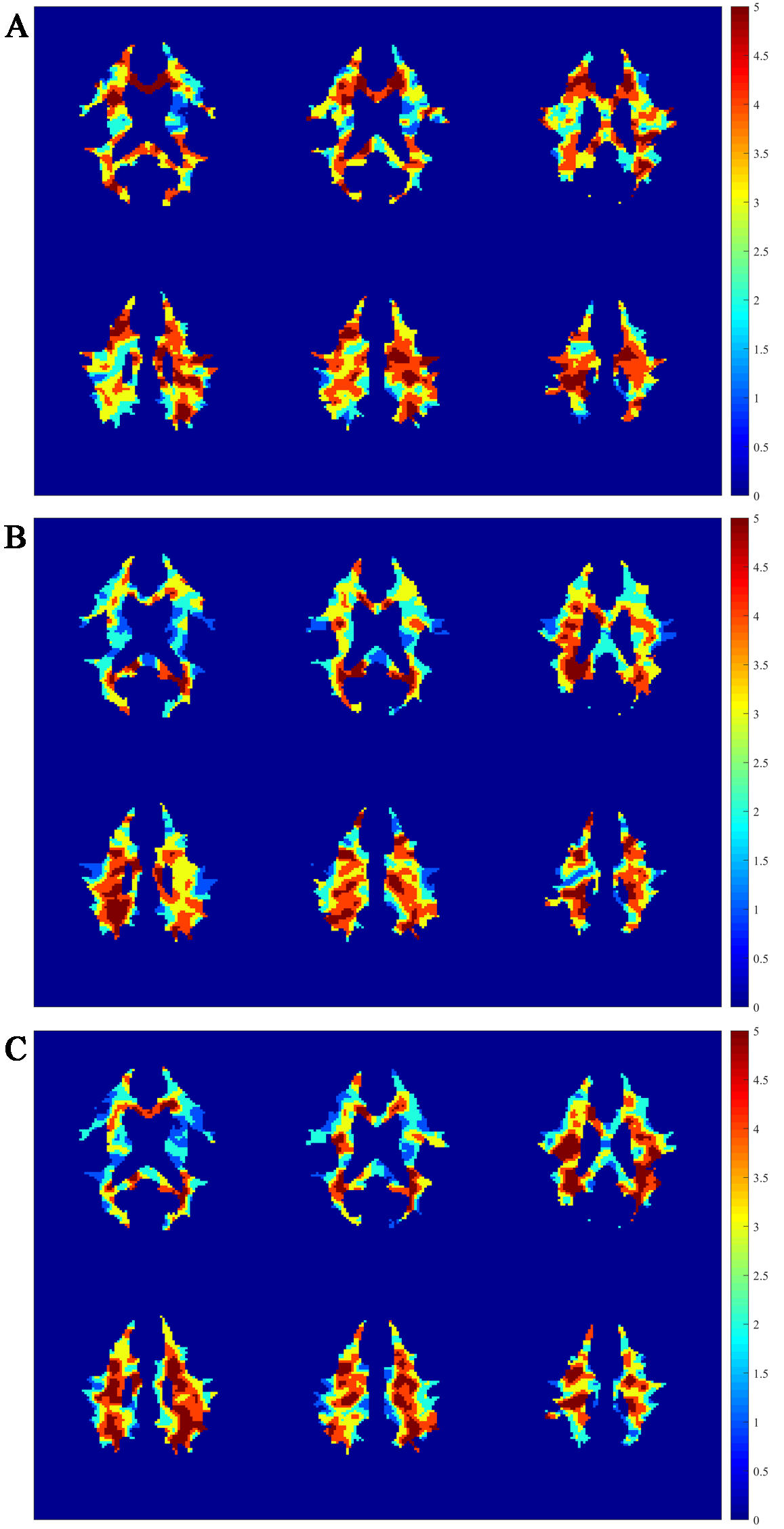


**Supplementary Fig. 4. (Continued)**


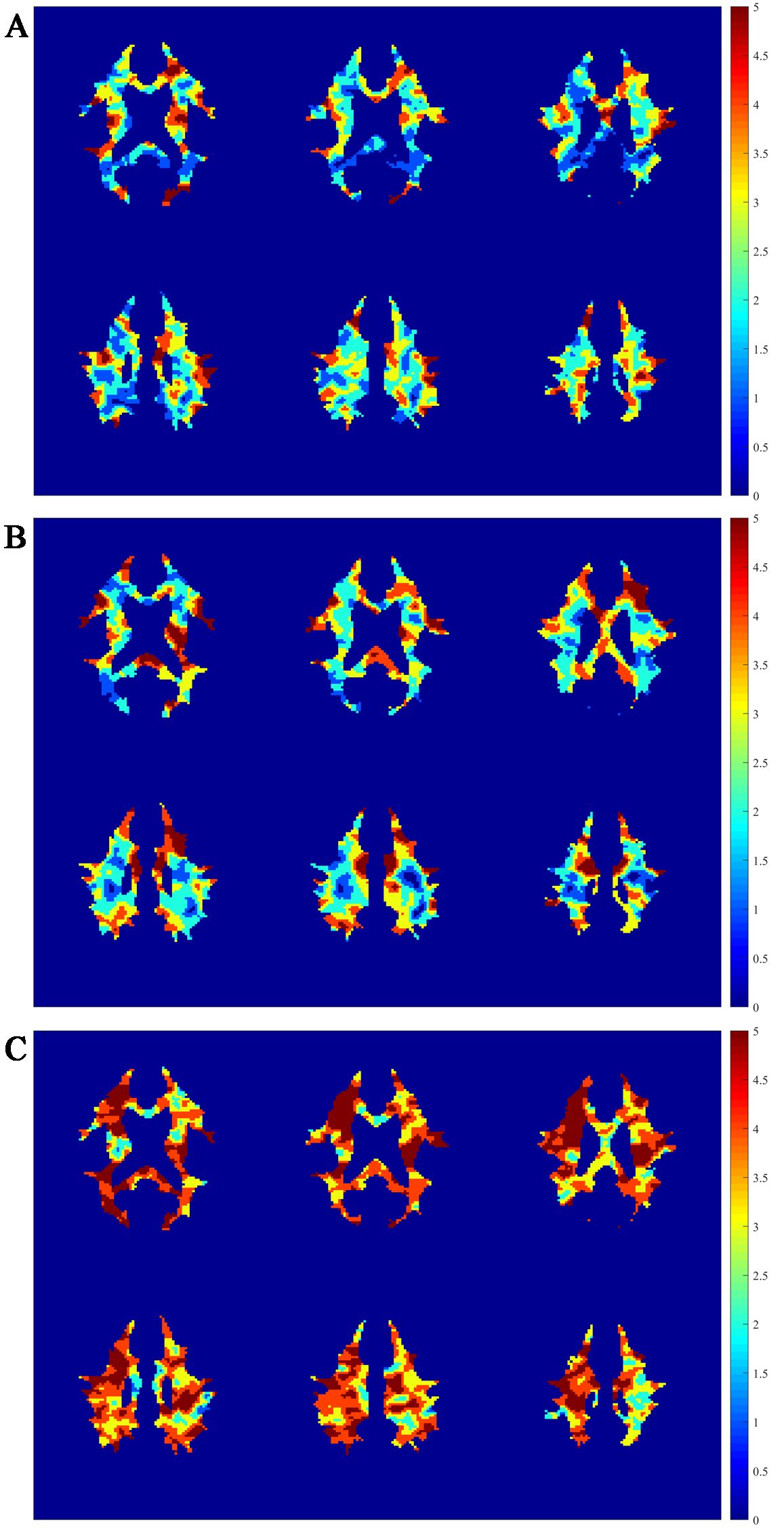


**Supplementary Fig. 4. (Continued)**


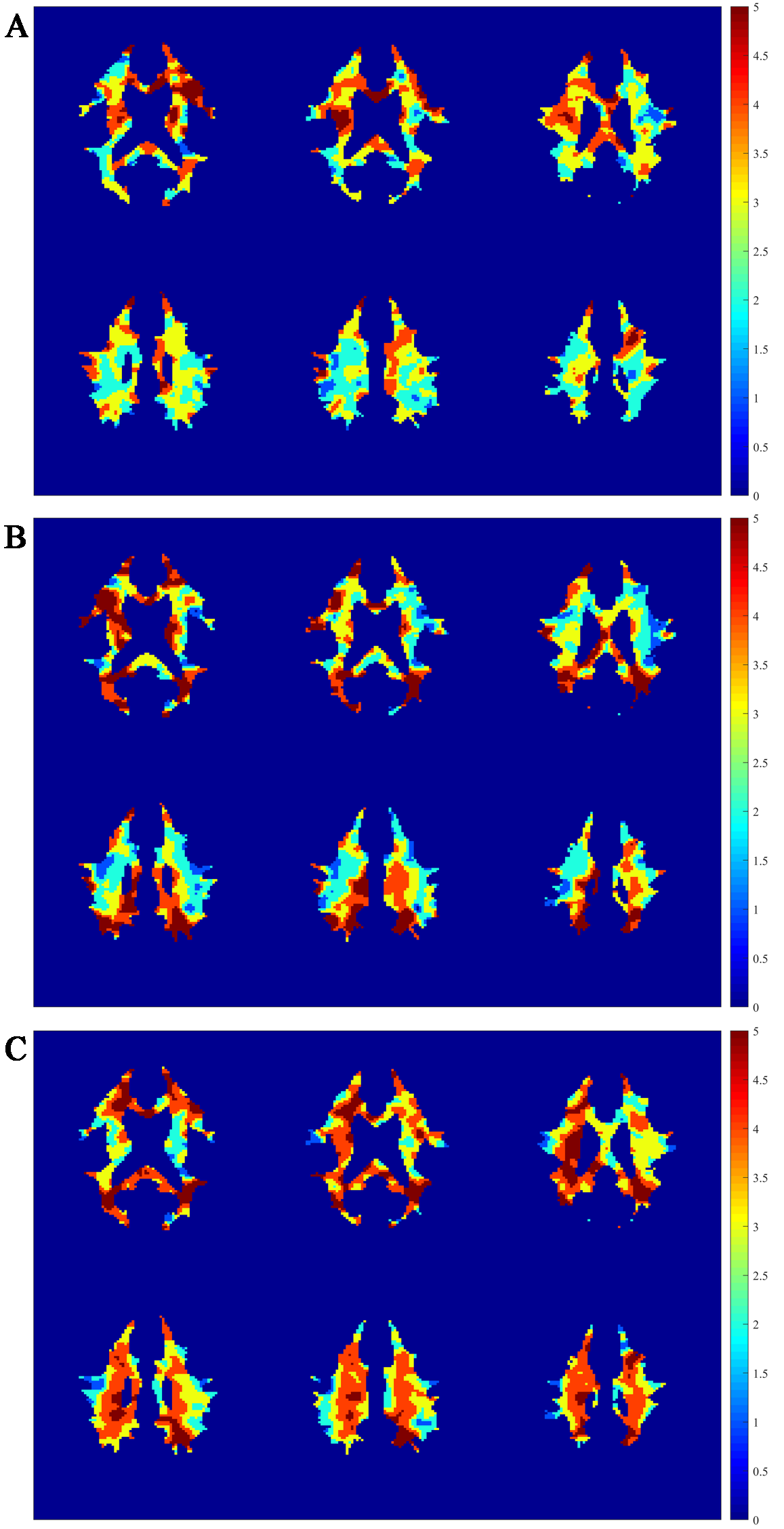


**Supplementary Fig. 4. (Continued)**


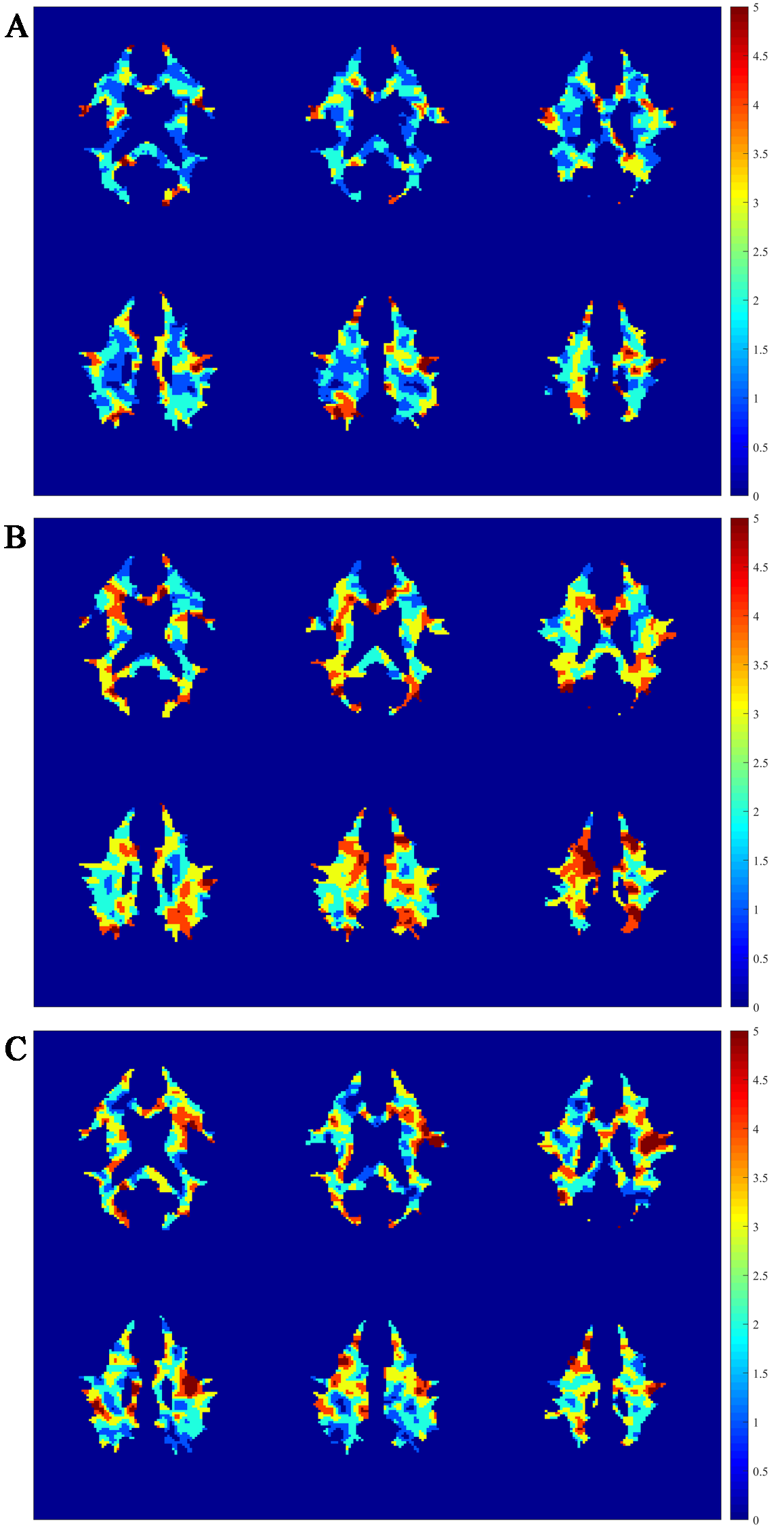


**Supplementary Fig. 4. (Continued)**


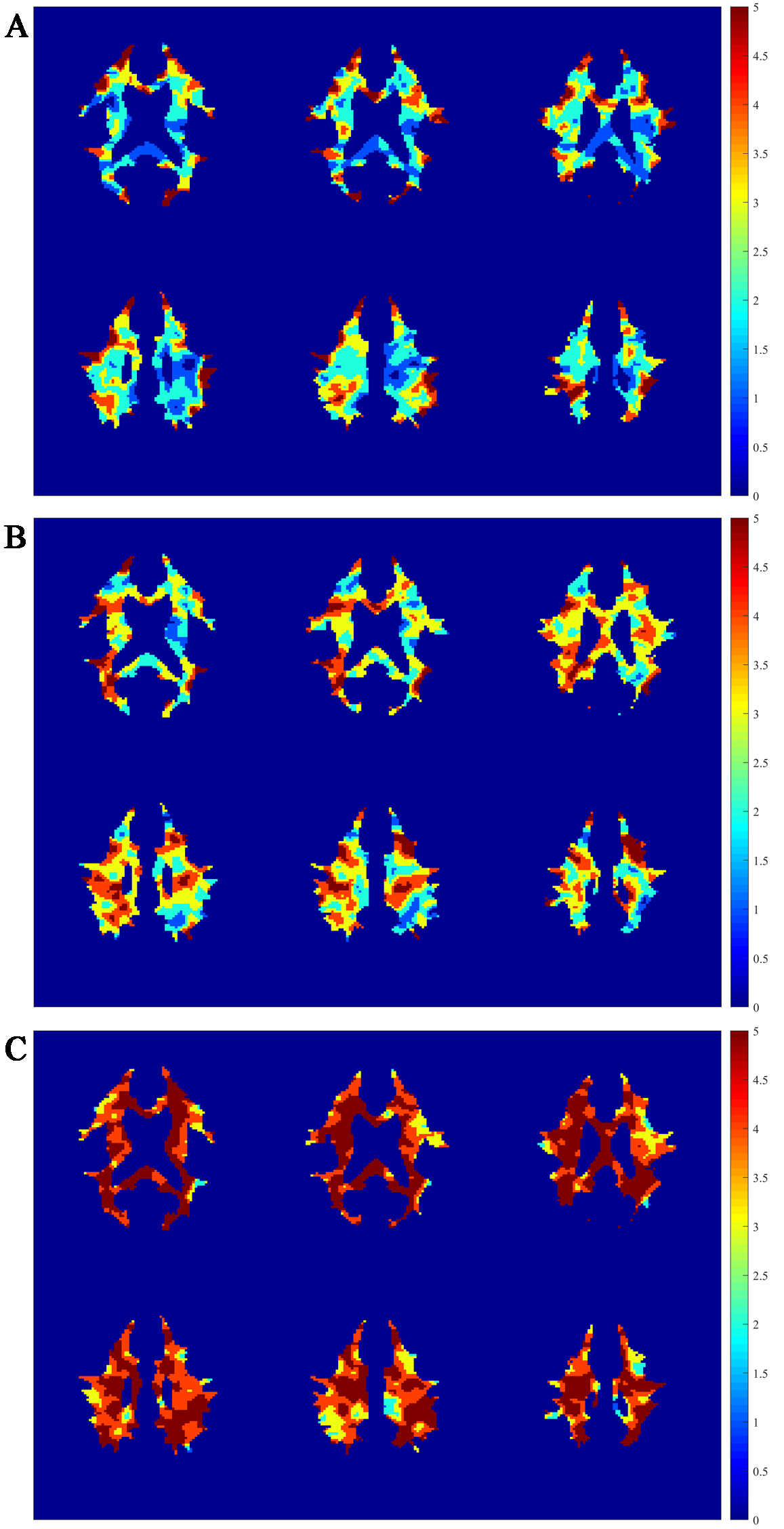


**Supplementary Fig. 4. (Continued)**


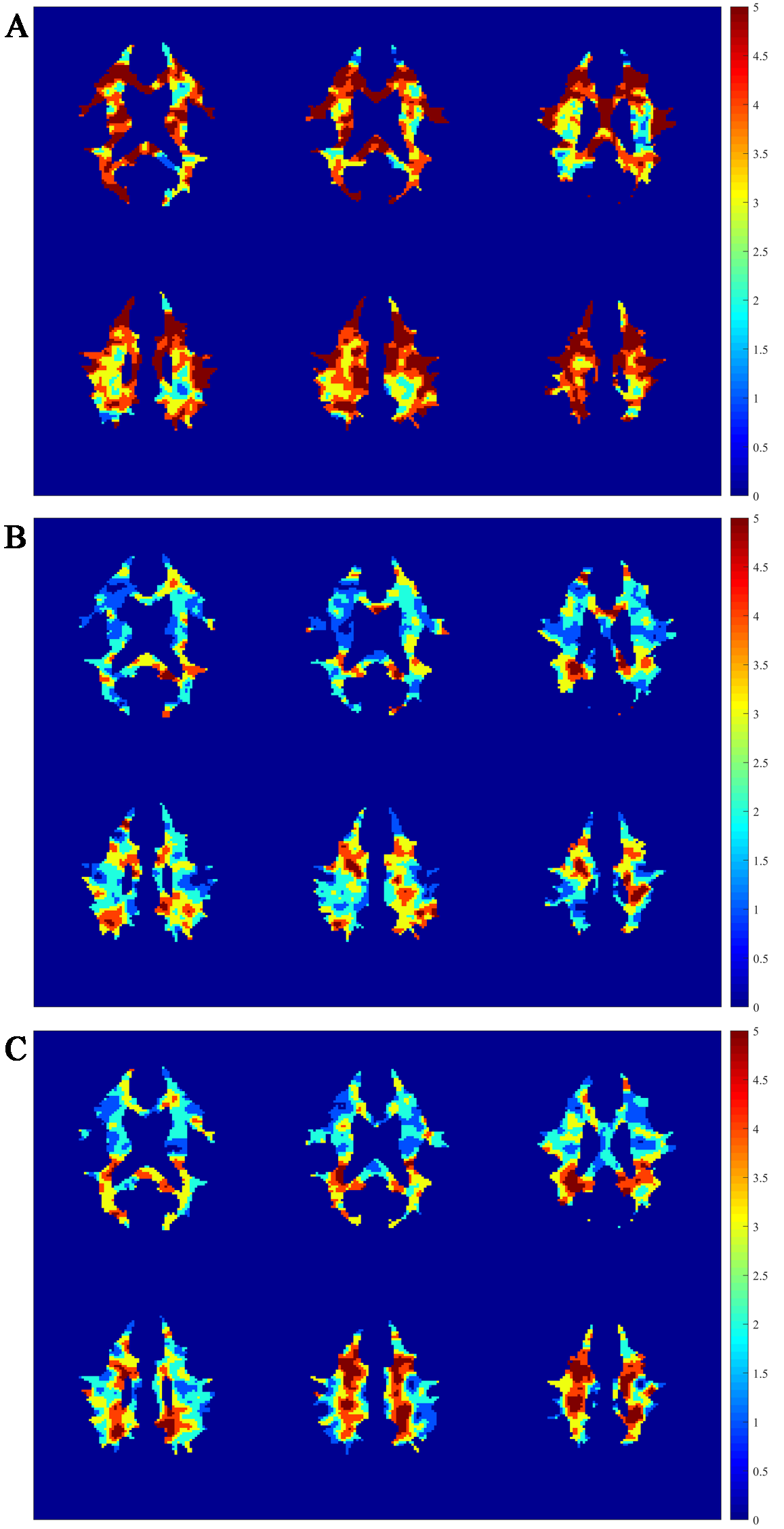


**Supplementary Fig. 4. (Continued)**


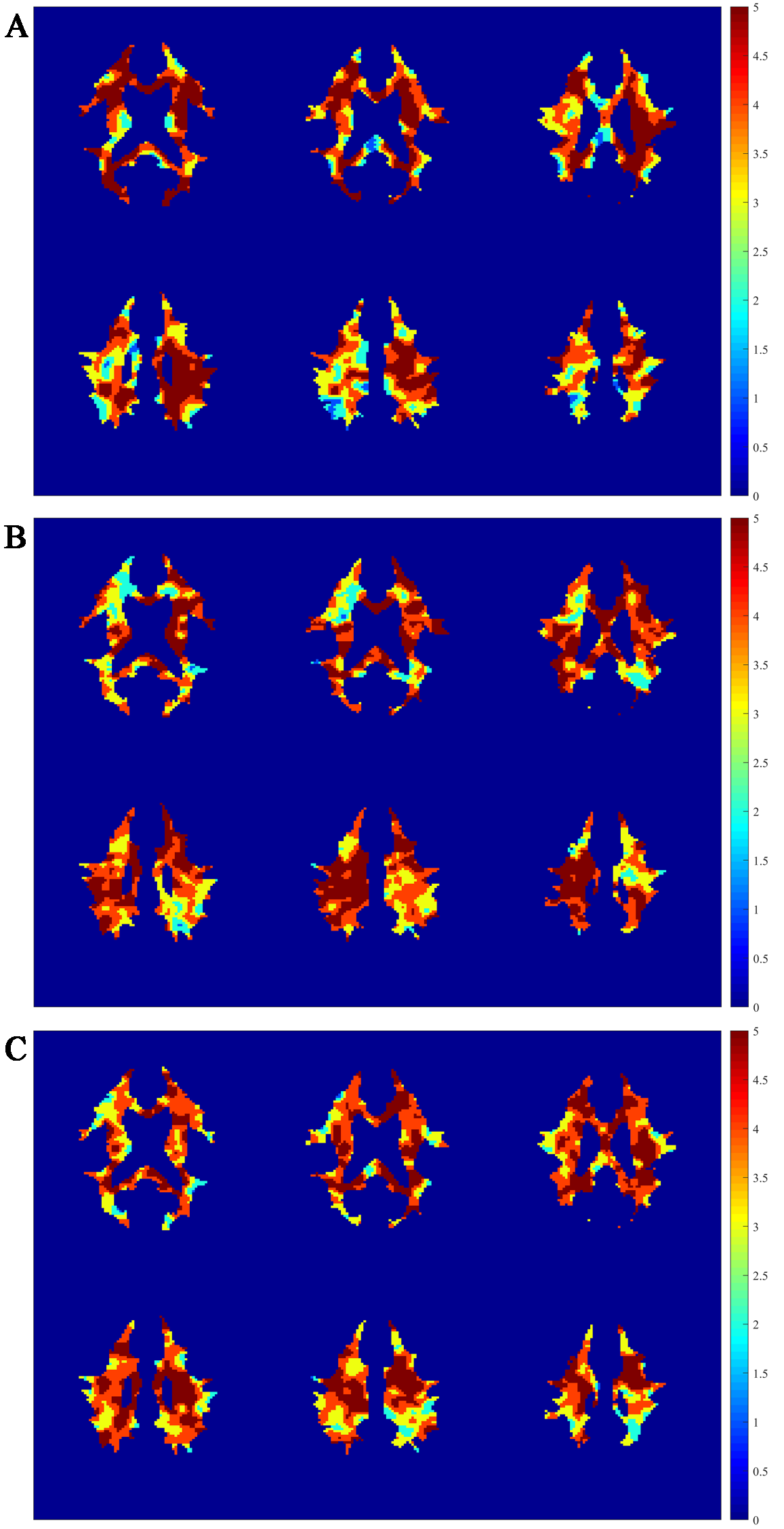


**Supplementary Fig. 4. (Continued)**


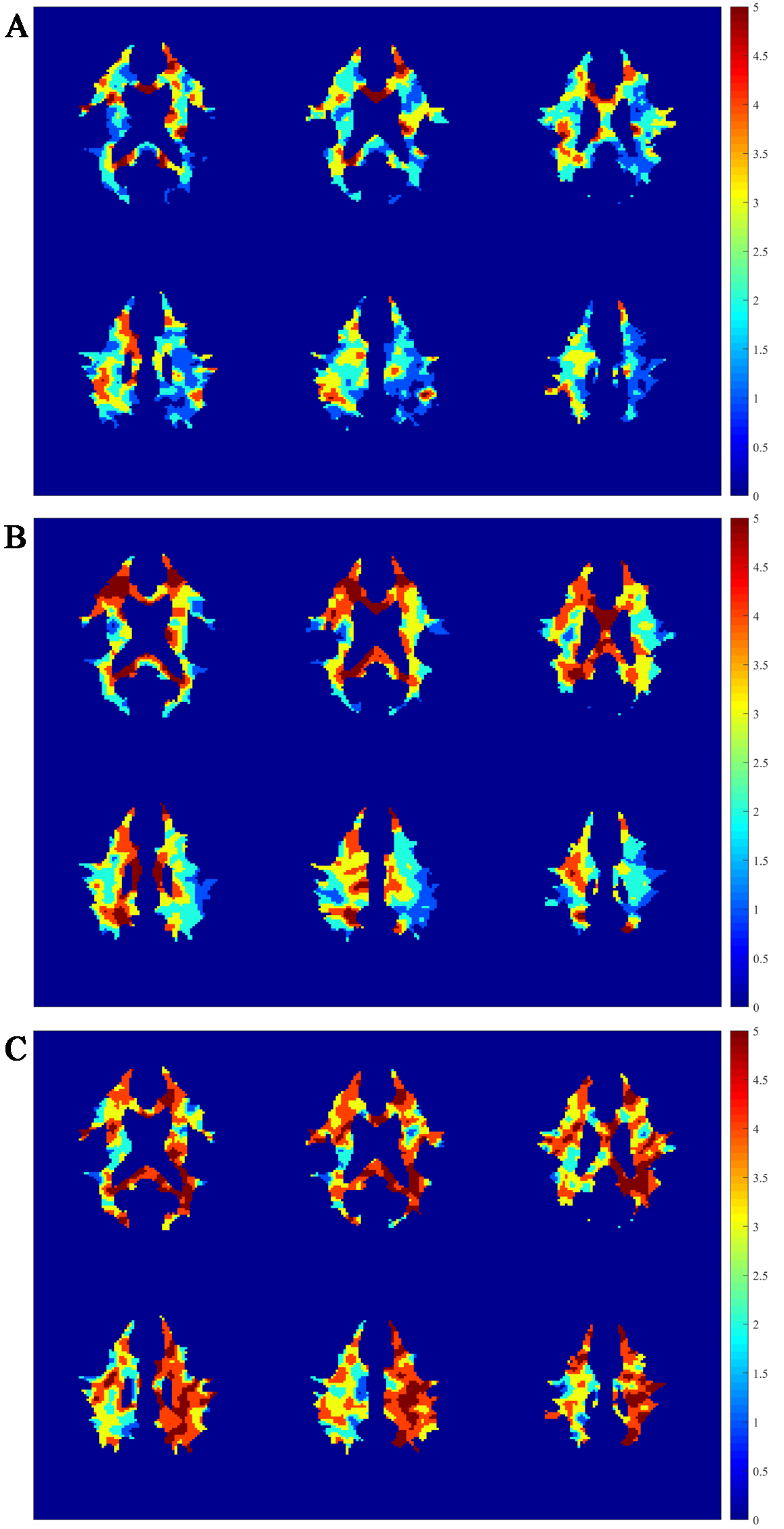


**Supplementary Fig. 4. (Continued)**


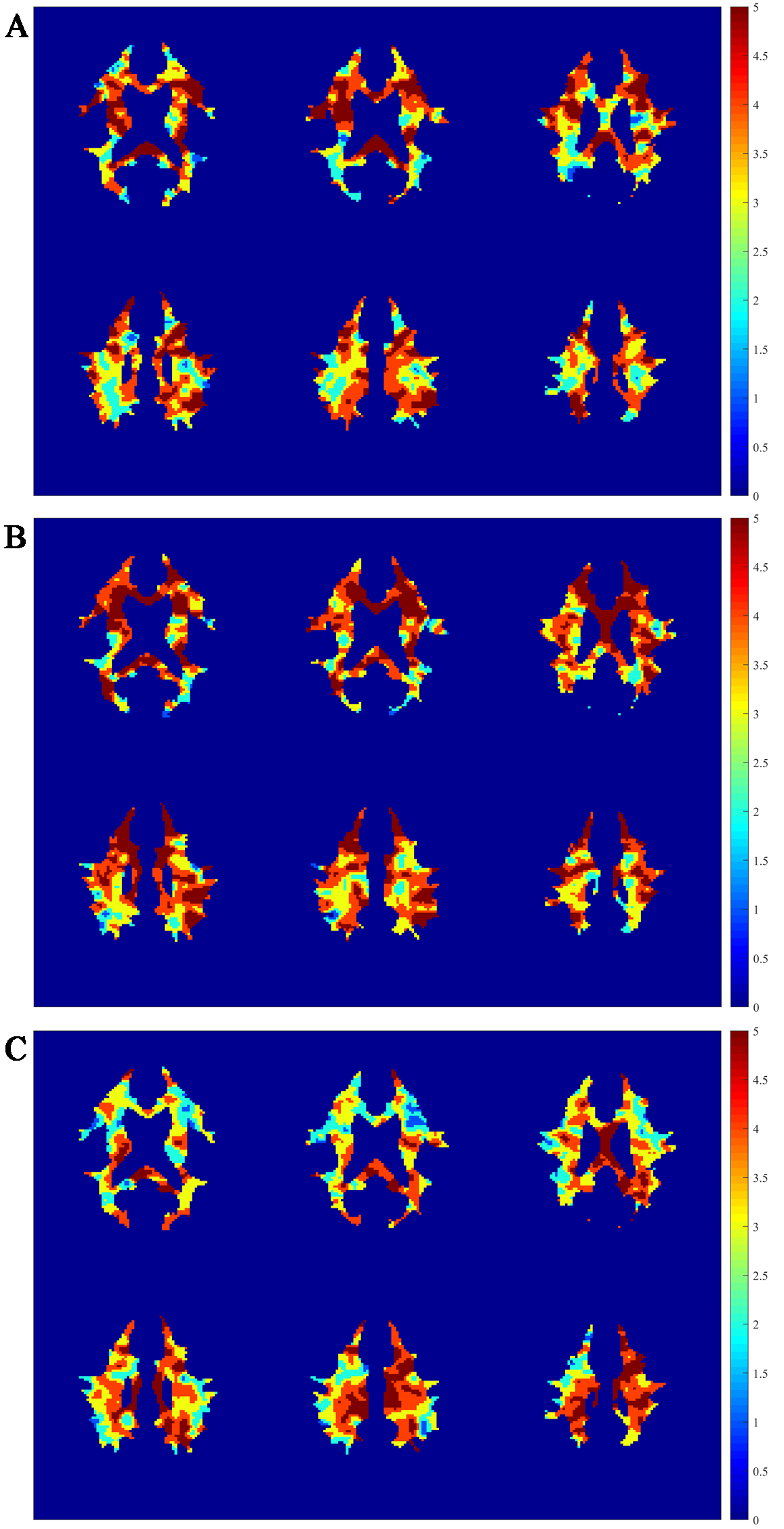


**Supplementary Fig. 4. (Continued)**


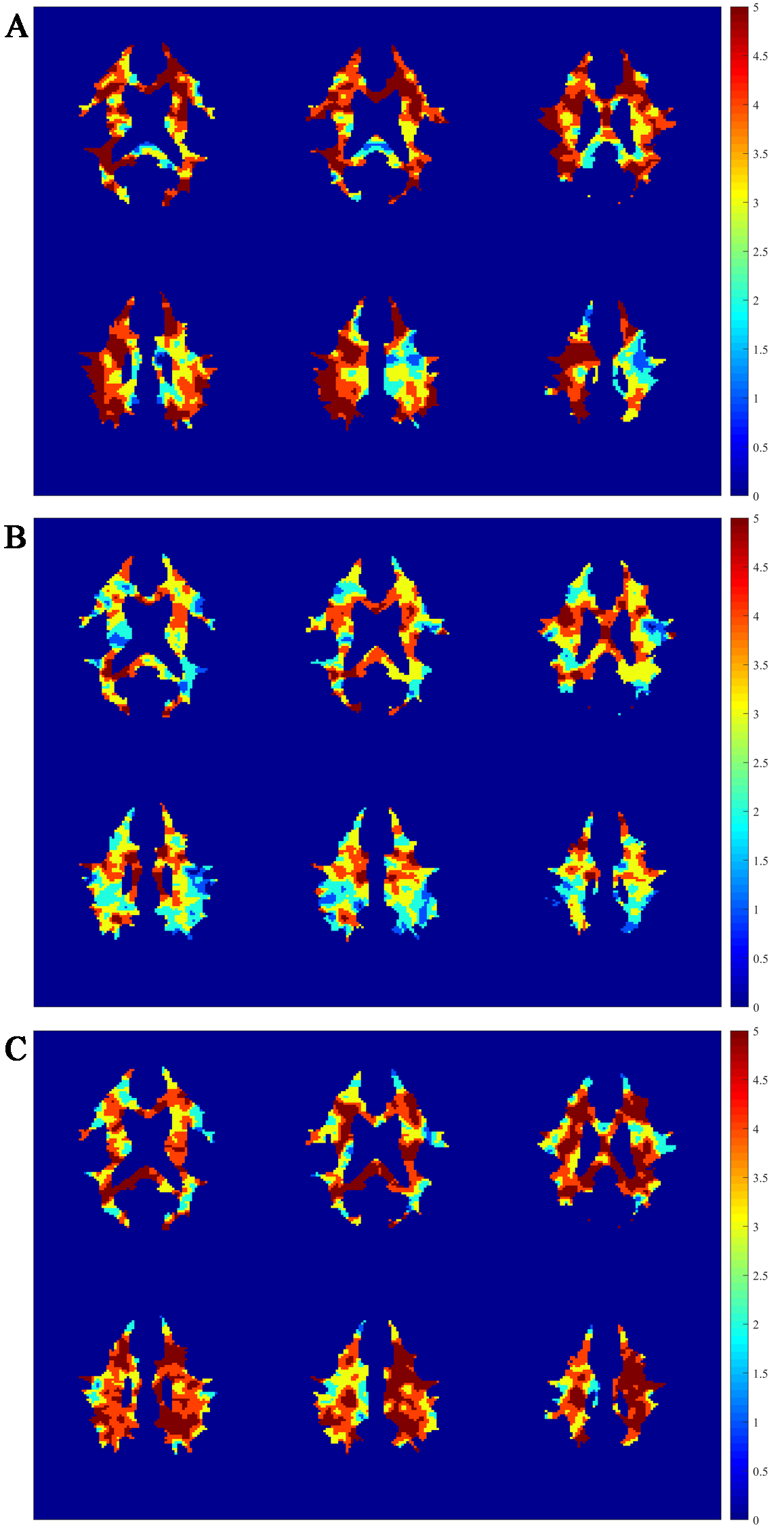


**Supplementary Fig. 4. (Continued)**


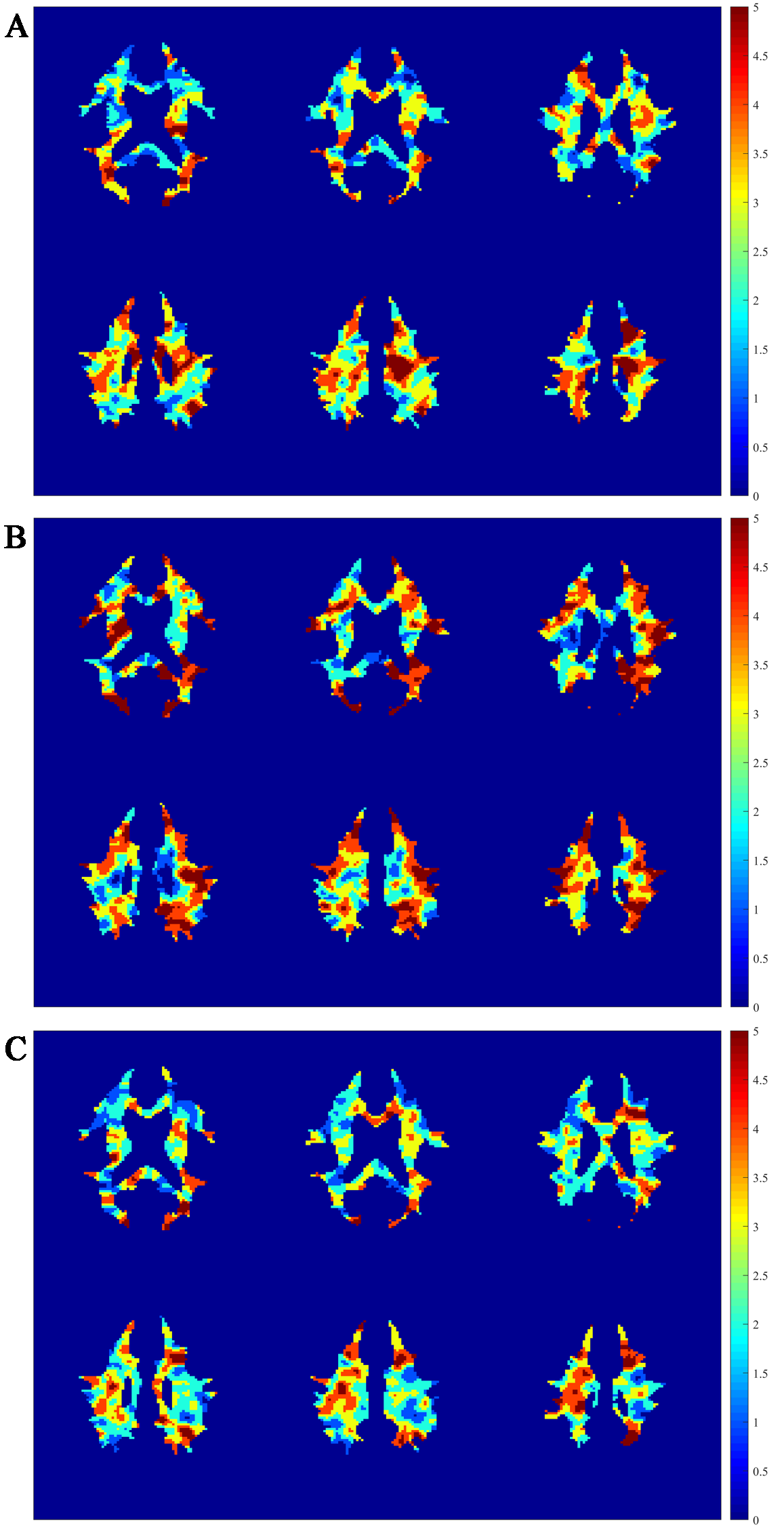


**Supplementary Fig. 4. (Continued)**


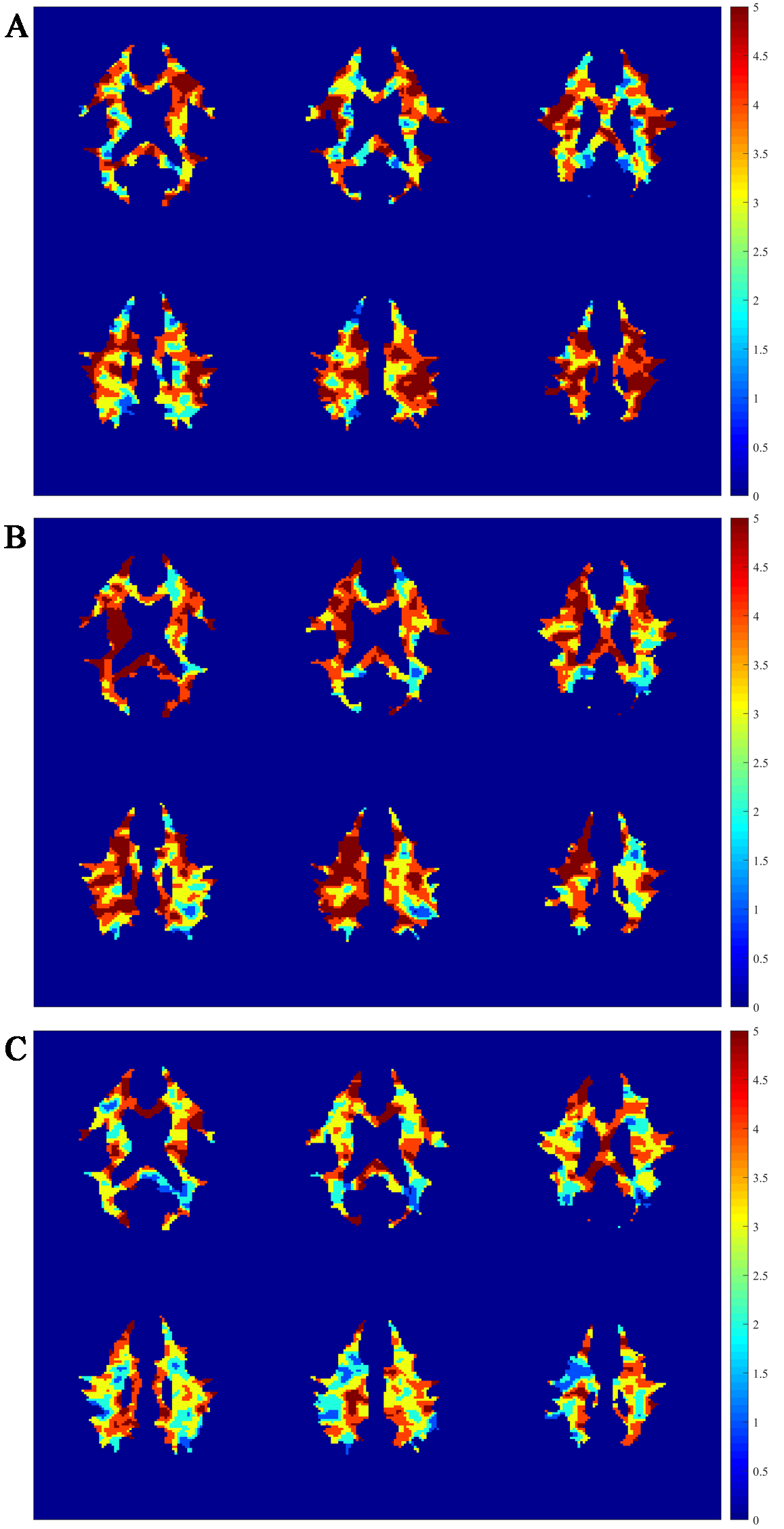


**Supplementary Fig. 4. (Continued)**


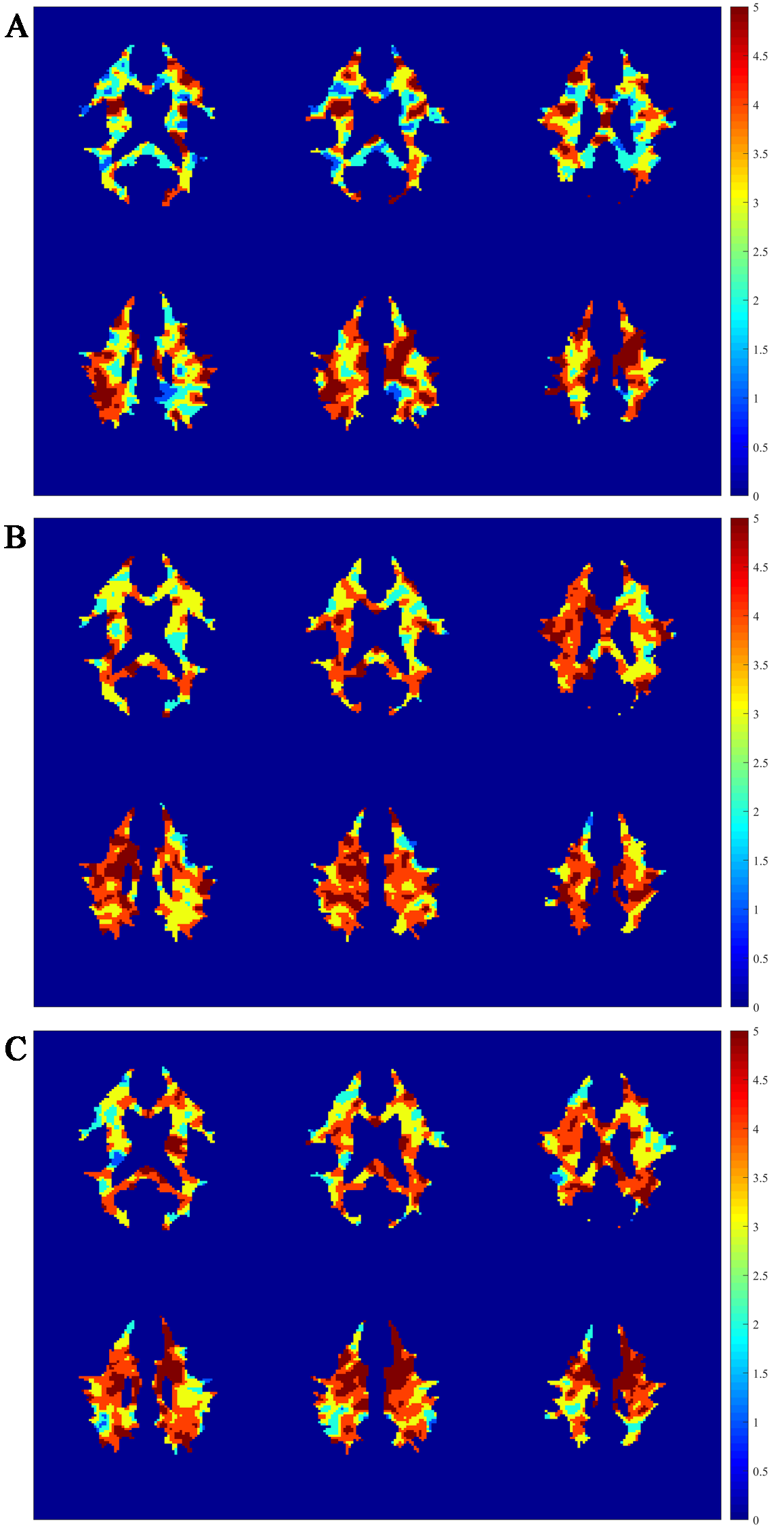


**Supplementary Fig. 4. (Continued)**


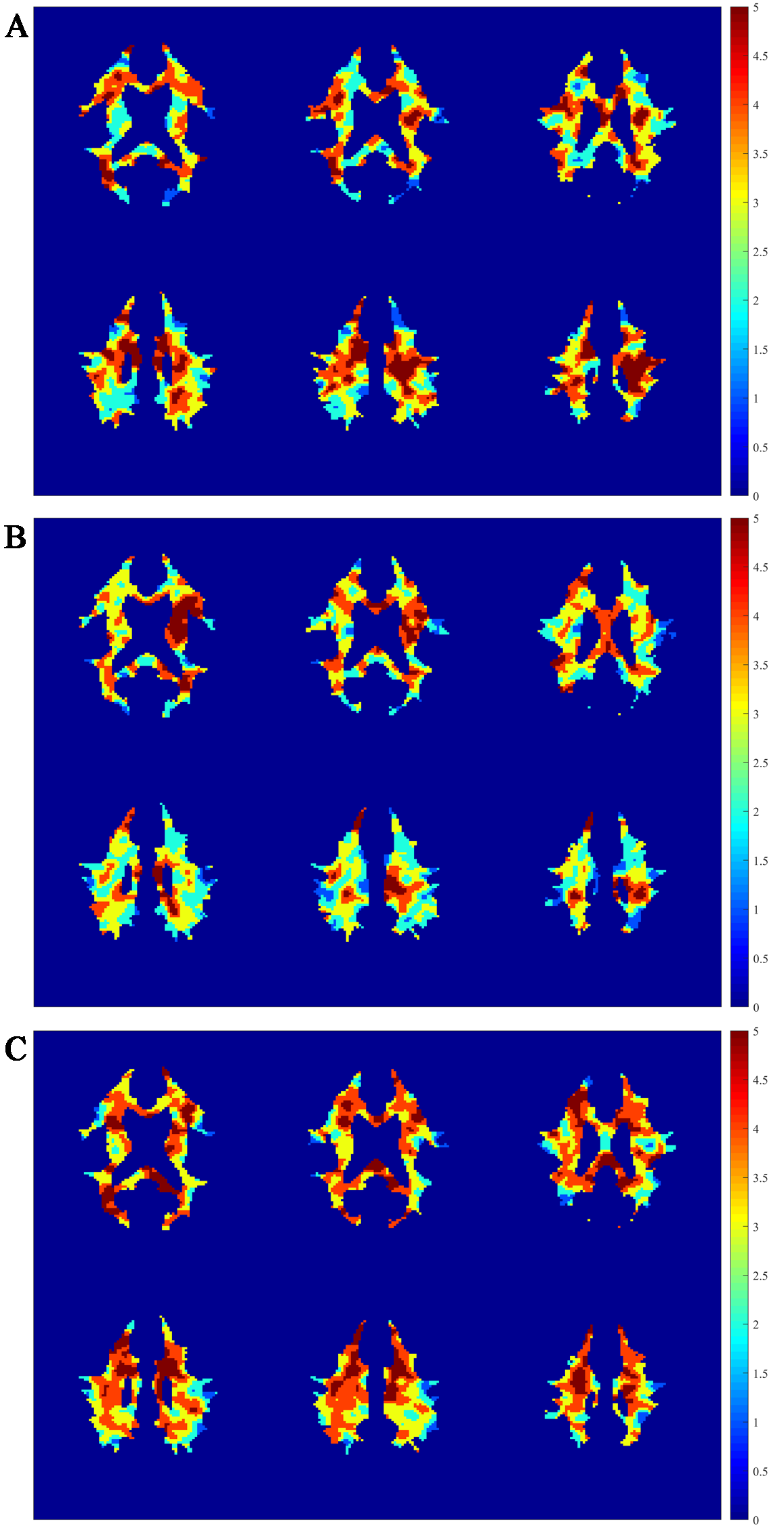


**Supplementary Fig. 4. (Continued)**


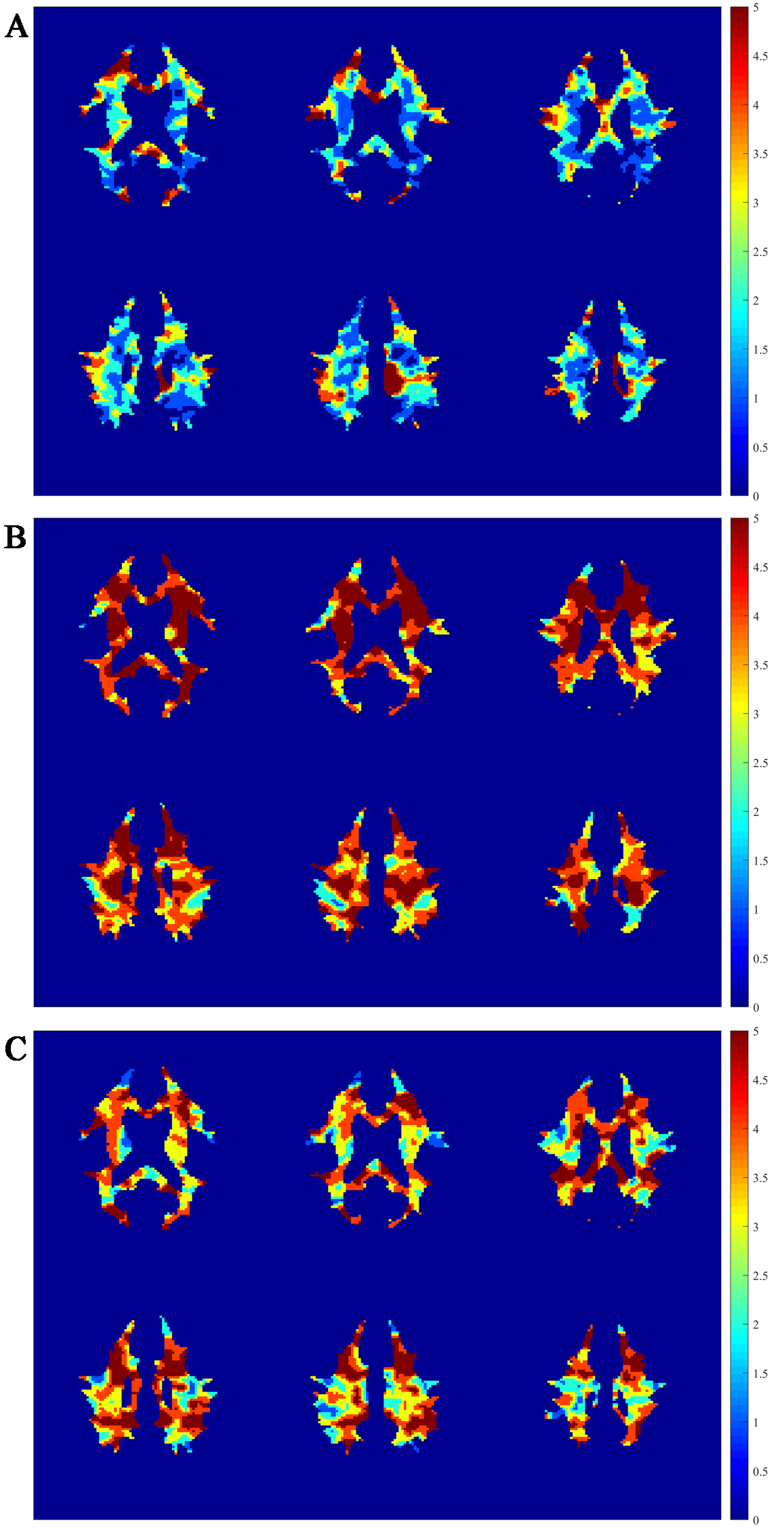


**Supplementary Fig. 4. (Continued)**


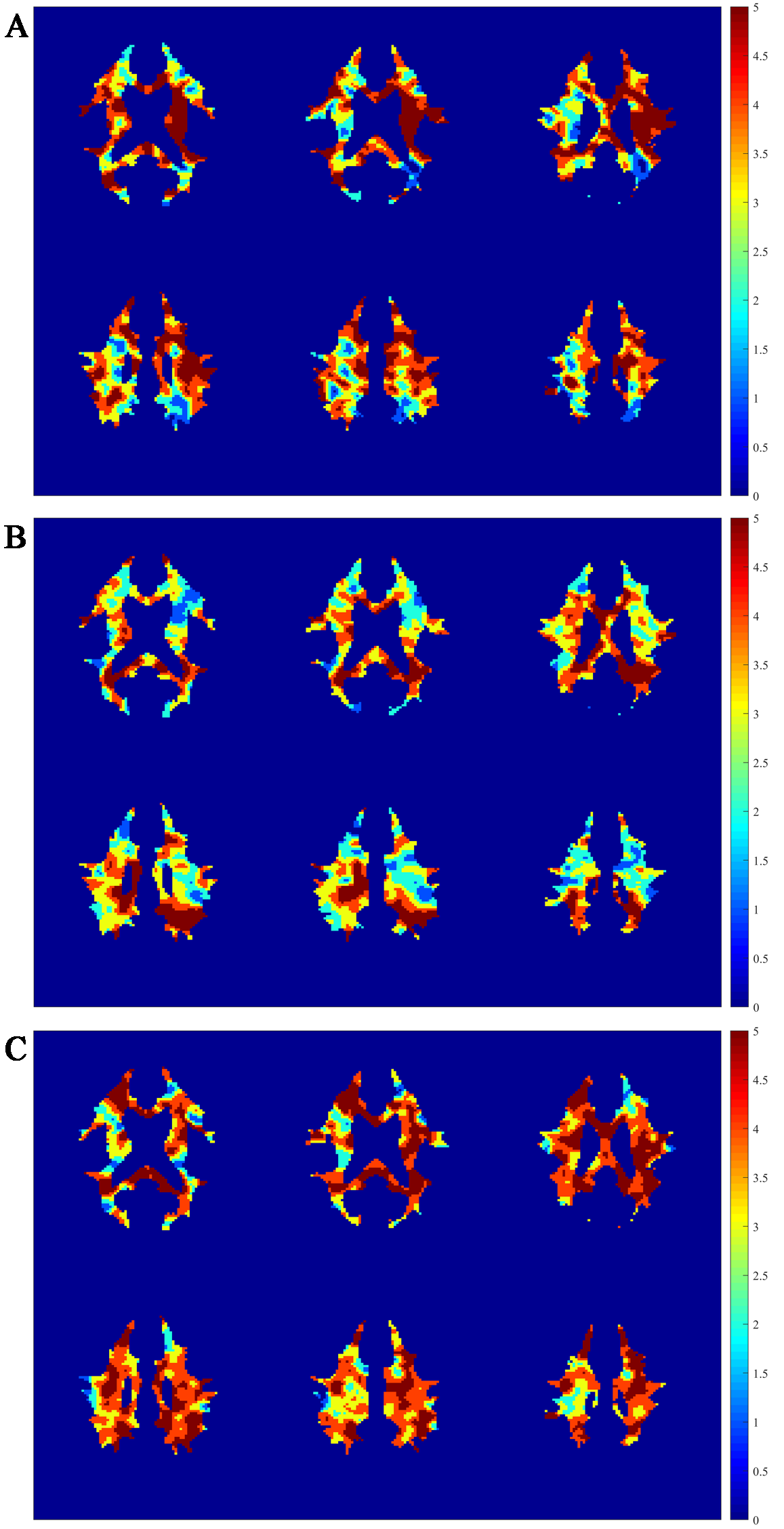


**Supplementary Fig. 4. (Continued)**


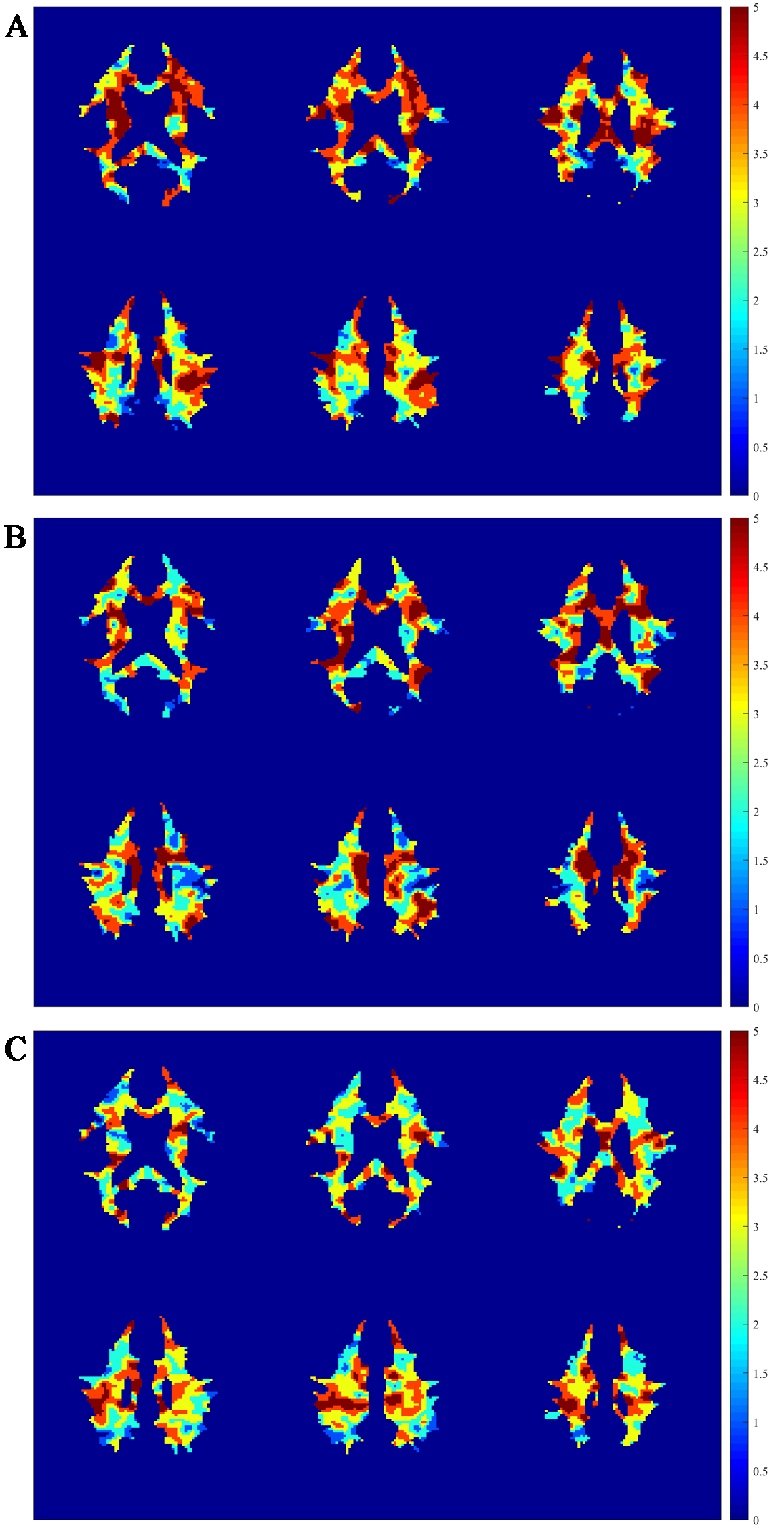


**Supplementary Fig. 4. (Continued)**


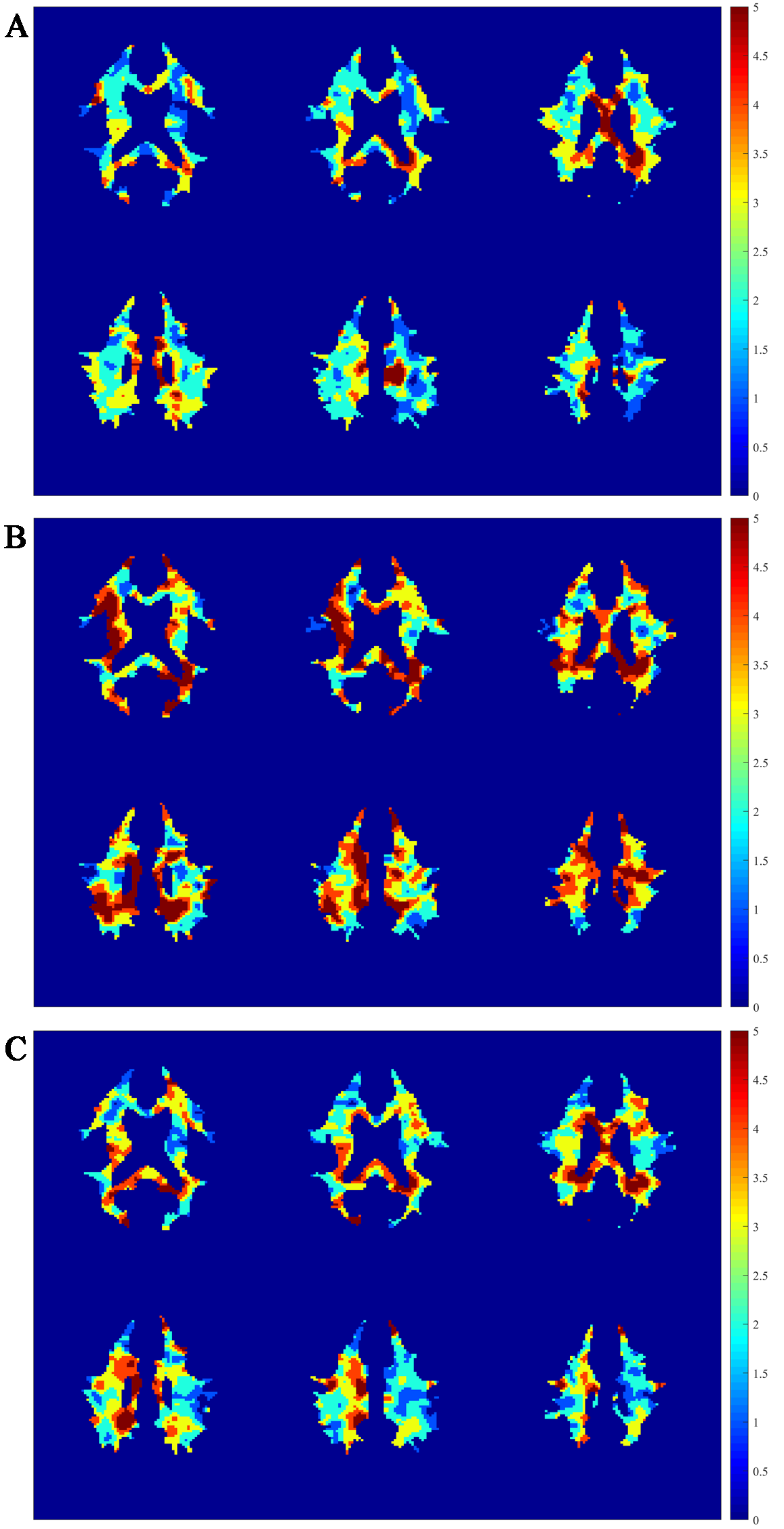


**Supplementary Fig. 4. (Continued)**


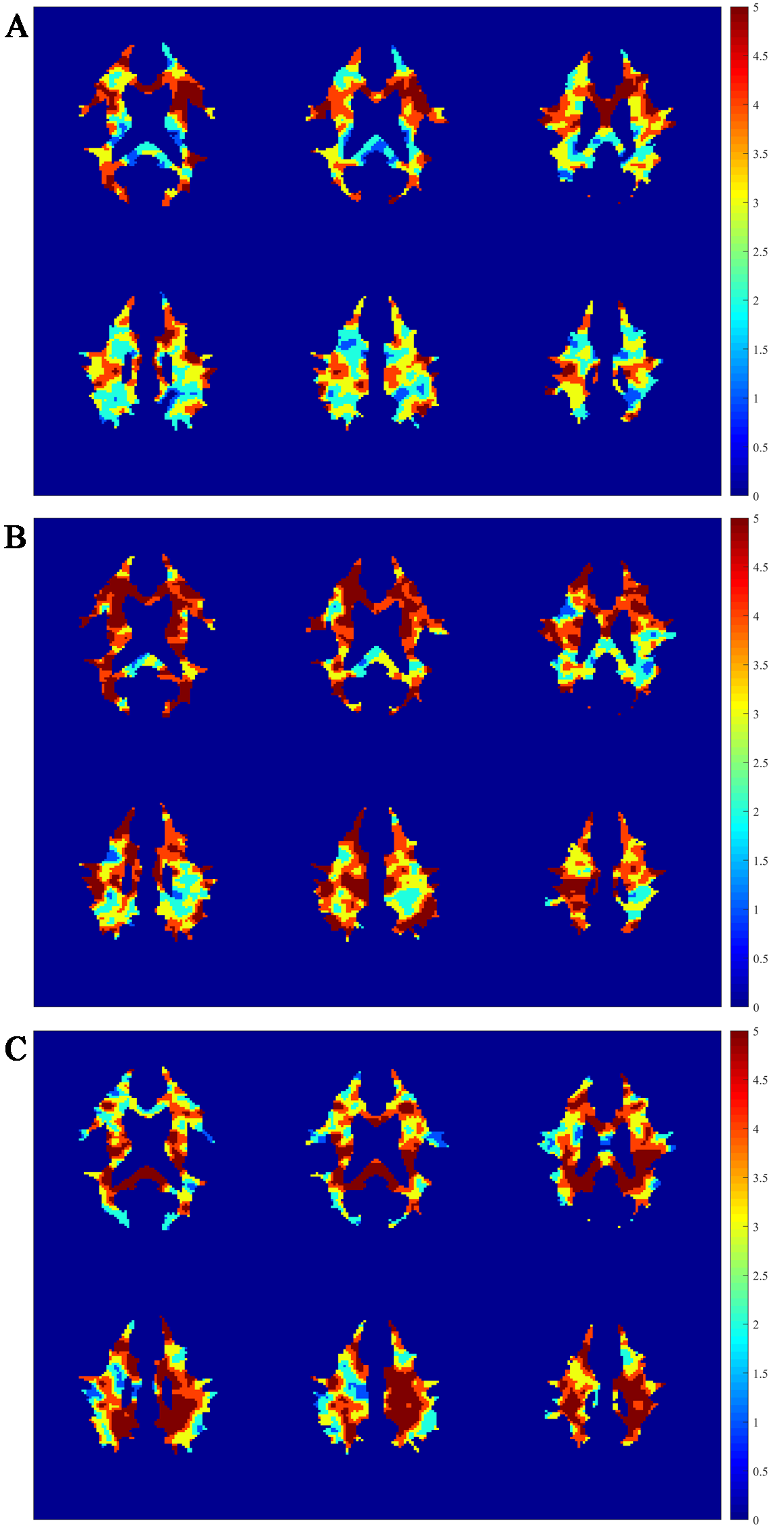


**Supplementary Fig. 4. (Continued)**


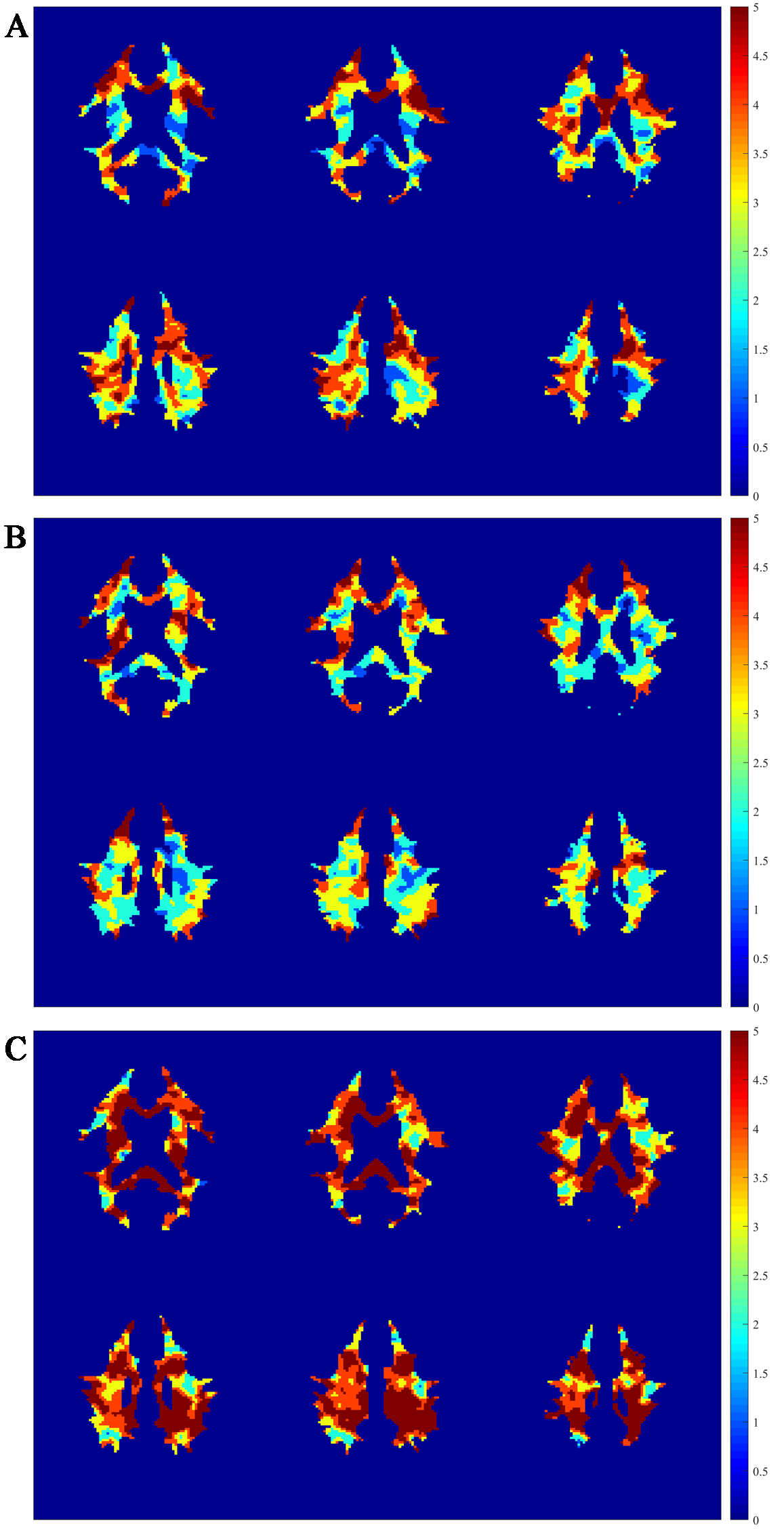


**Supplementary Fig. 4. (Continued)**


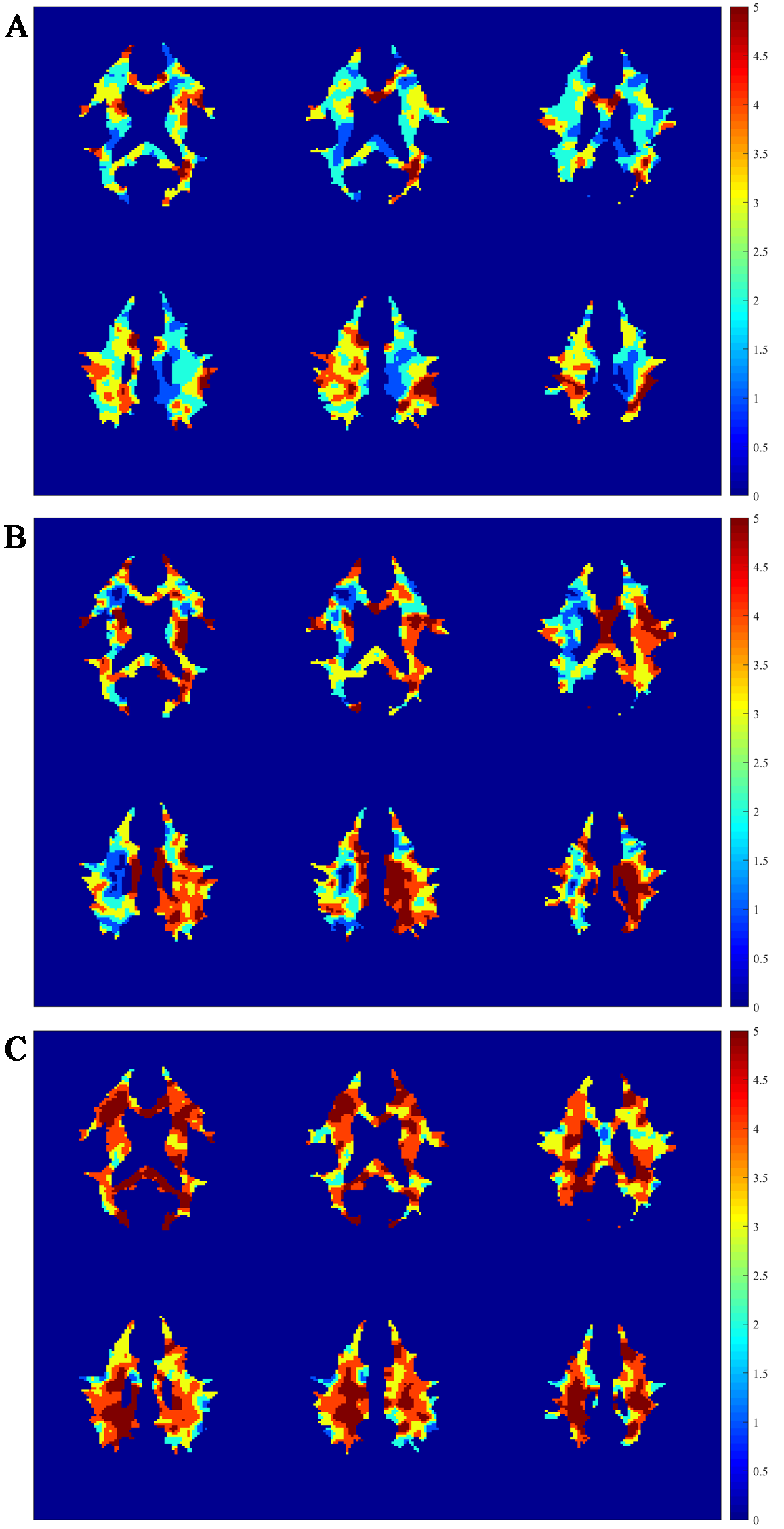


**Supplementary Fig. 4. (Continued)**


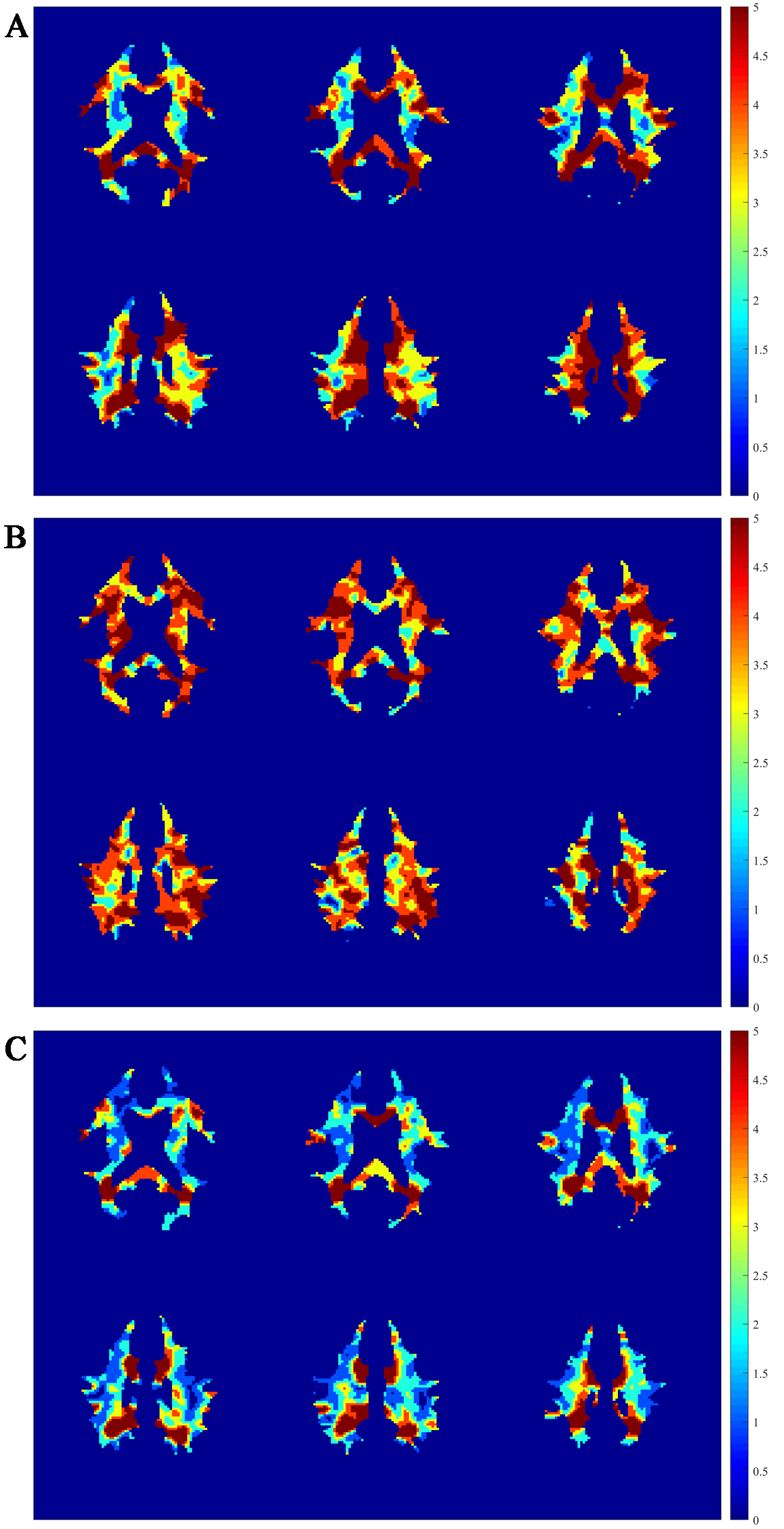


**Supplementary Fig. 4. (Continued)**


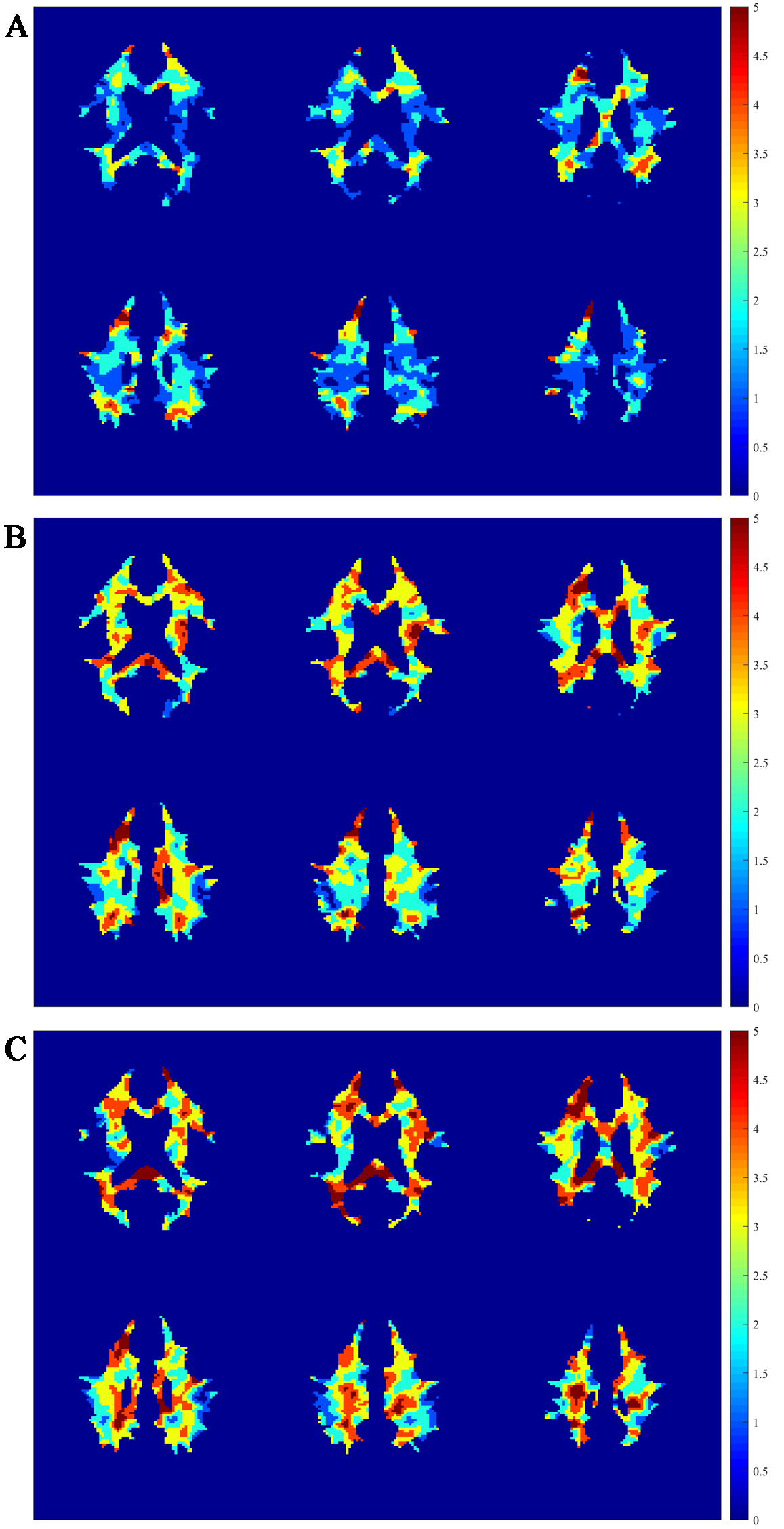


**Supplementary Fig. 4. (Continued)**


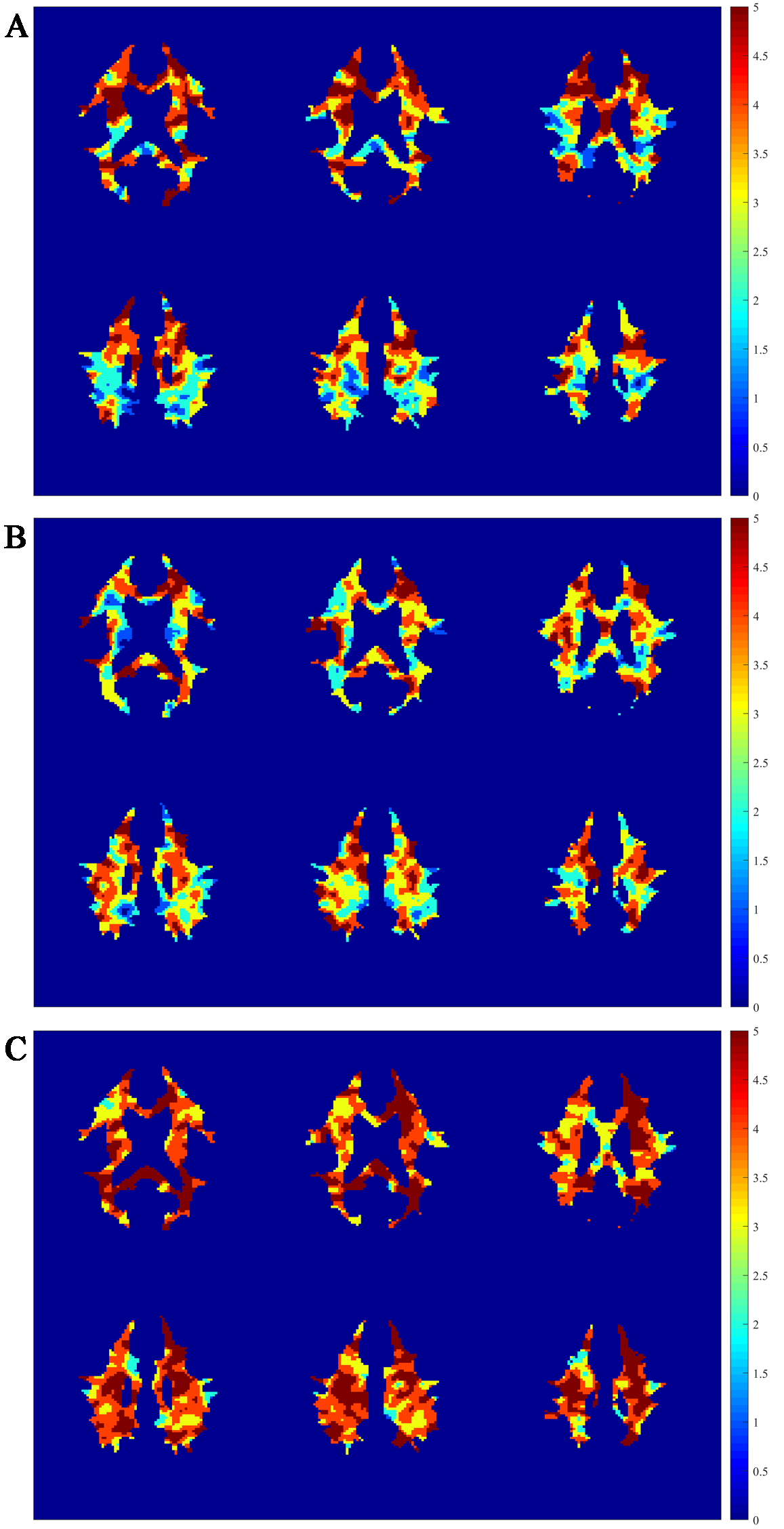


**Supplementary Fig. 4. (Continued)**


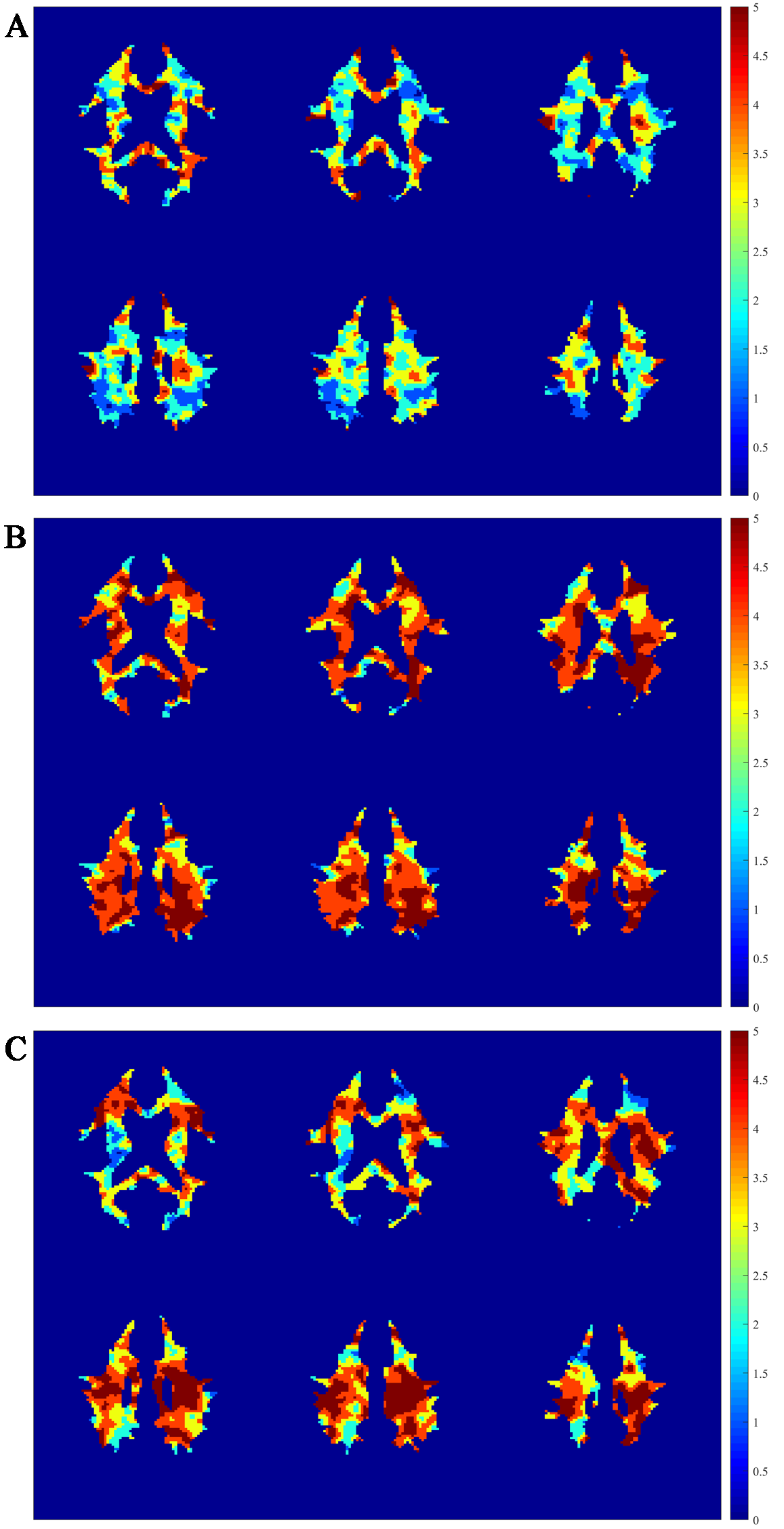


**Supplementary Fig. 4. (Continued)**


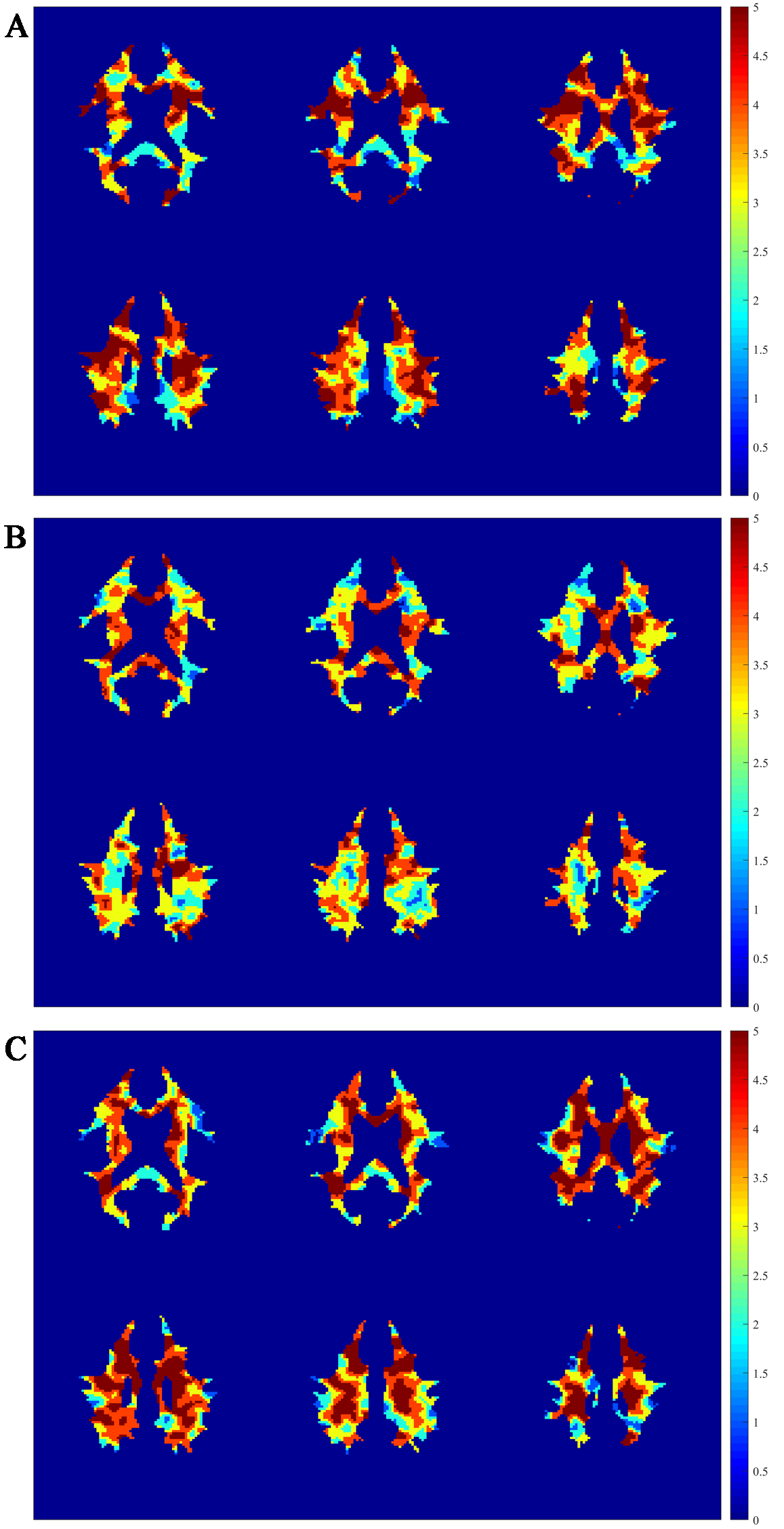


**Supplementary Fig. 4. (Continued)**


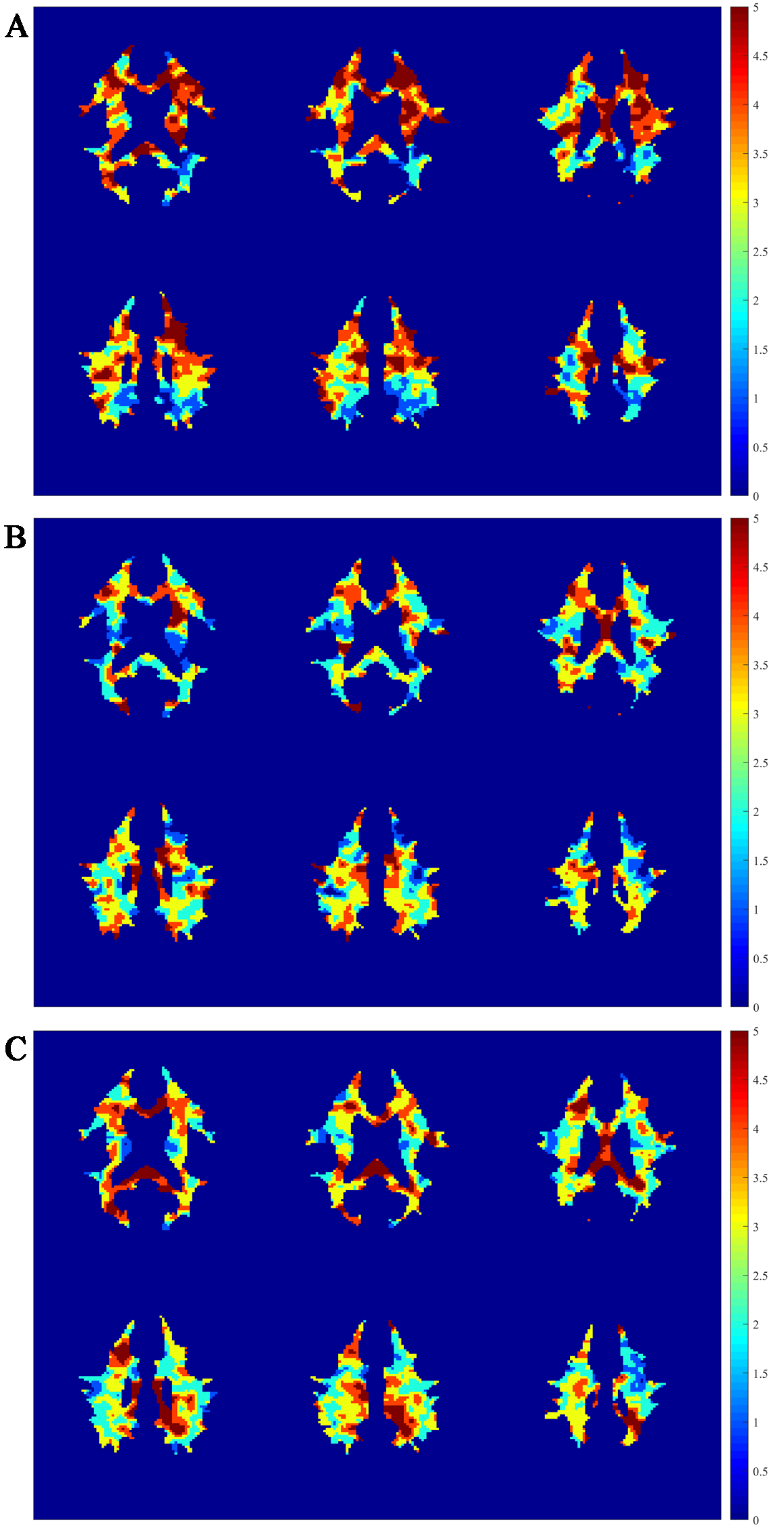


**Supplementary Fig. 4. (Continued)**


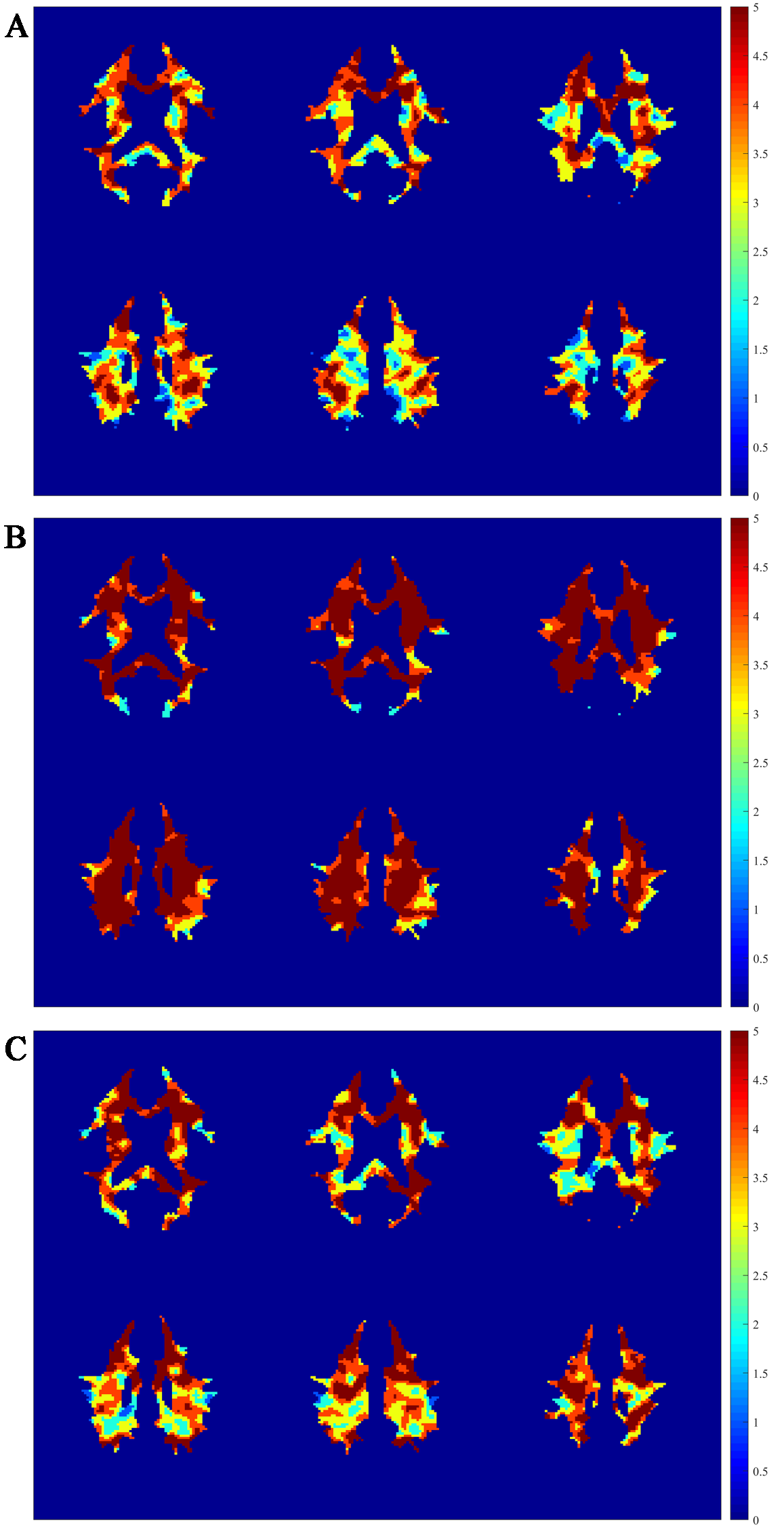


**Supplementary Fig. 4. (Continued)**


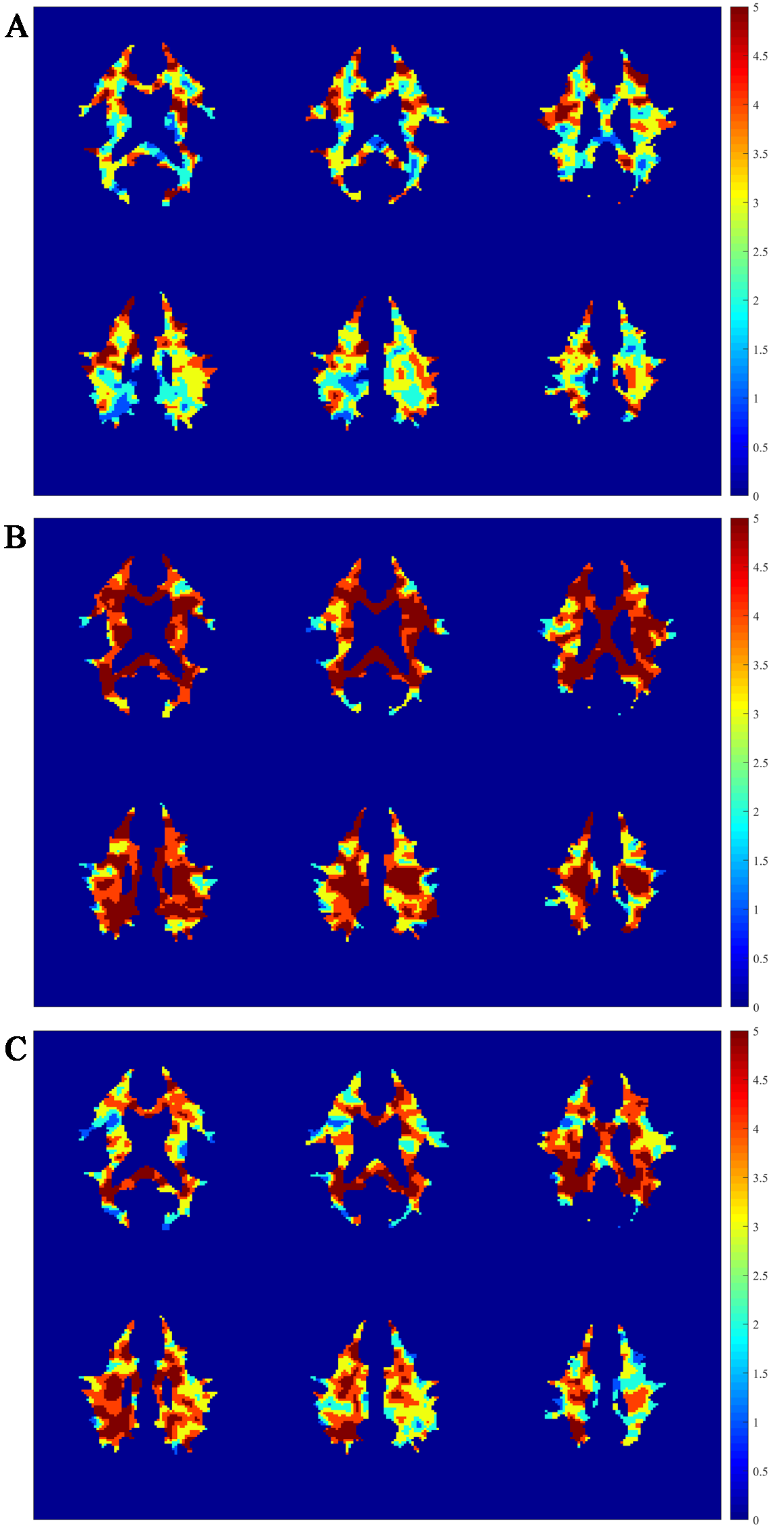


**Supplementary Fig. 4. (Continued)**


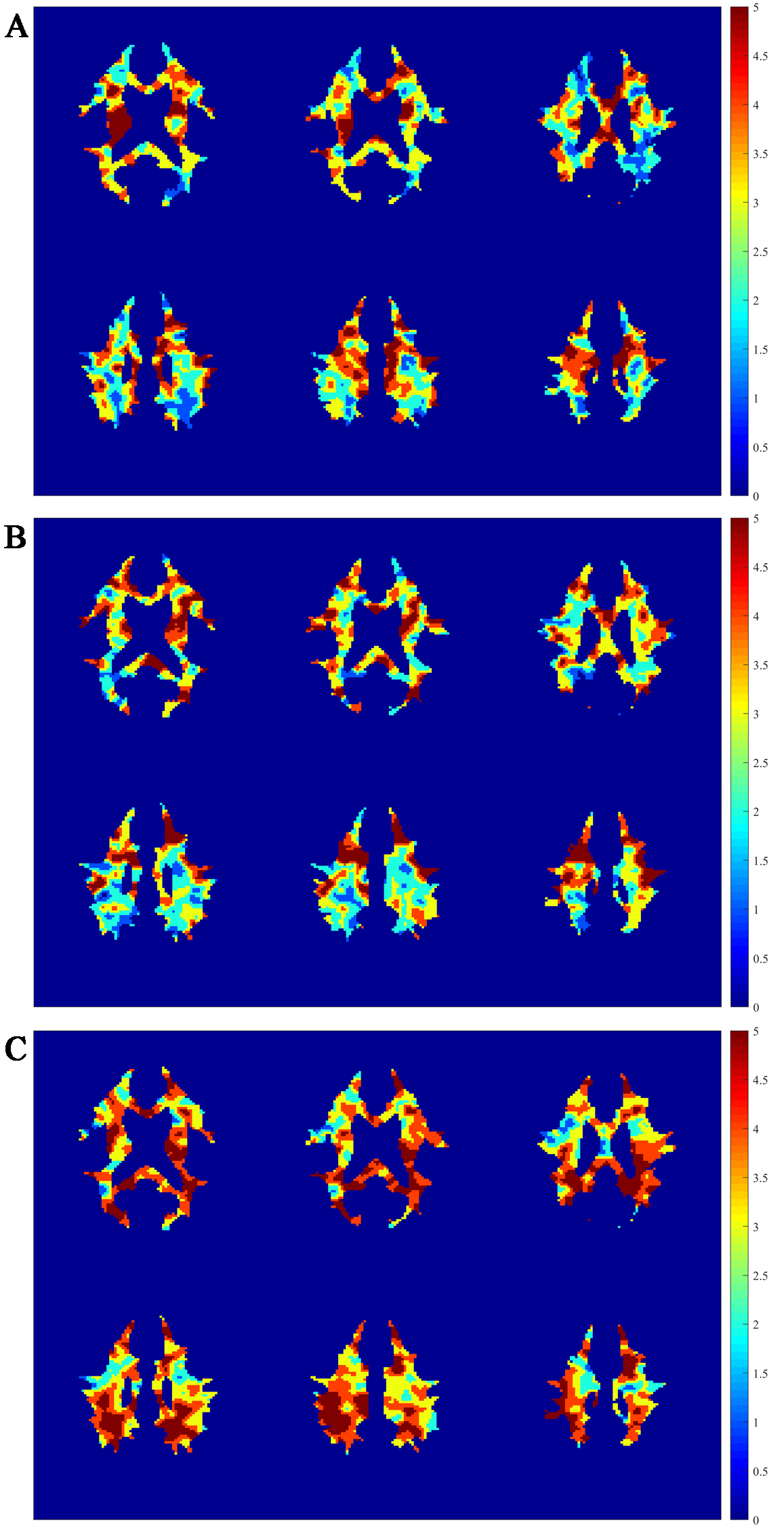


**Supplementary Fig. 4. (Continued)**


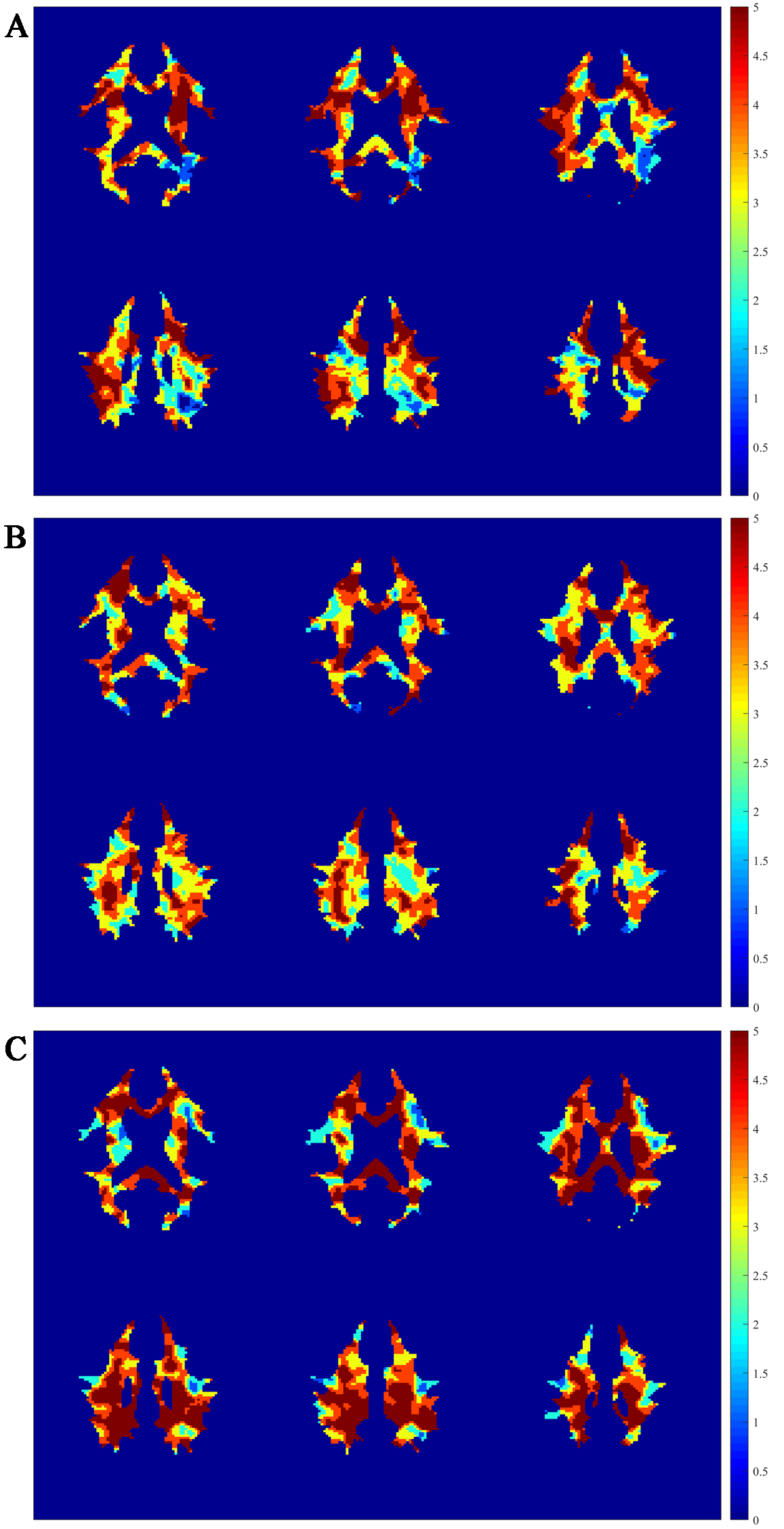


**Supplementary Fig. 4. (Continued)**


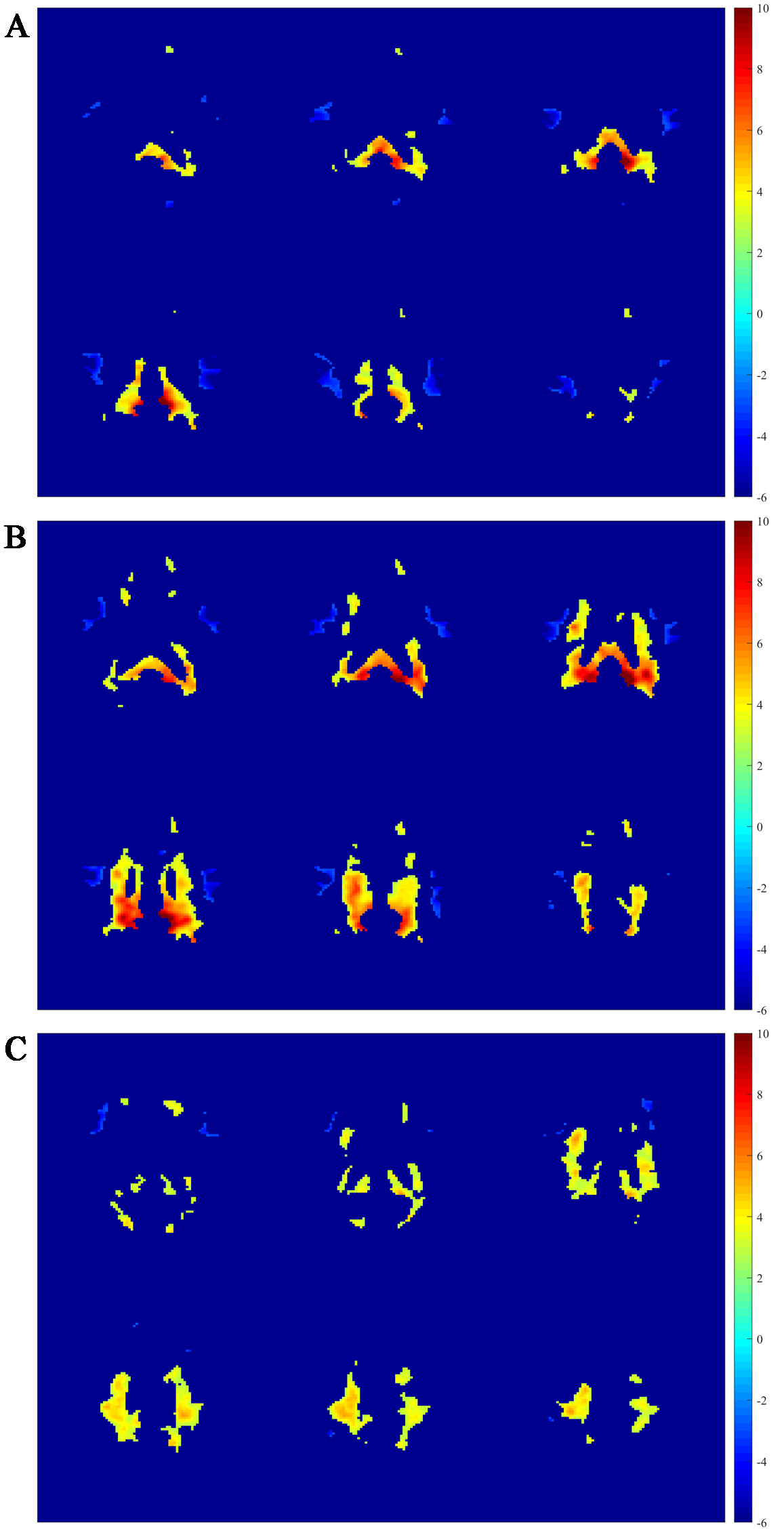


**Supplementary Fig. 5.** Maps of T-statistics of WM correlation coefficients among the three GM regions (paired t-test, p < 0.01, uncorrected). (A) Maps of T-statistic of WM correlation coefficients between left PCC and the left IPS. (B) Maps T-statistic of WM correlation coefficients between the left PCC and the right IFGoperc. (C) Maps of T-statistic of WM correlation coefficients between the left IPS and the right IFGoperc. The color bar denotes T-value.


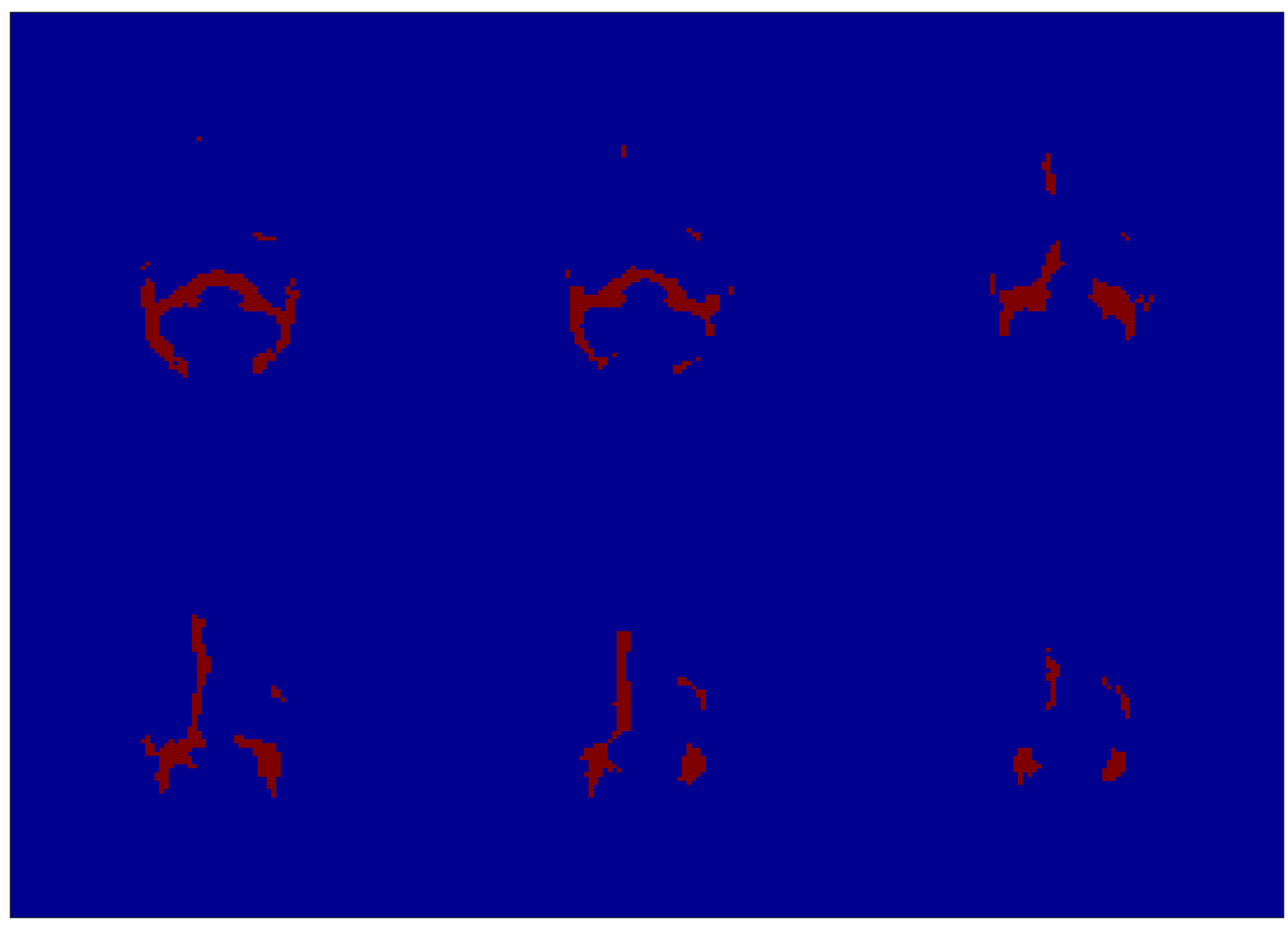


**Supplementary Fig. 6.** WM structural connectivity to the left PCC using probabilistic fiber tracking. For each subject, the probability map was confined within the WM using the WM mask and then binarized with a threshold (number of voxels in the seed mask × 5000 × 0.005). The final connectivity map was obtained by summing up individual binarized maps and thresholding it at > 75% of the subject account.
